# Efficient single-component white light emitting diodes enabled by lanthanide ions doped lead halide perovskites via controlling Förster energy transfer and specific defect clearance

**DOI:** 10.1038/s41377-022-01027-9

**Published:** 2022-12-06

**Authors:** Rui Sun, Donglei Zhou, Yujiao Ding, Yue Wang, Yuqi Wang, Xinmeng Zhuang, Shuainan Liu, Nan Ding, Tianyuan Wang, Wen Xu, Hongwei Song

**Affiliations:** State Key Laboratory of Integrated Optoelectronics, College of Electronic Science and Engineering, Jilin University, Changchun, 130012 China

**Keywords:** Inorganic LEDs, Quantum dots

## Abstract

Currently, a major challenge for metal-halide perovskite light emitting diodes (LEDs) is to achieve stable and efficient white light emission due to halide ion segregation. Herein, we report a promising method to fabricate white perovskite LEDs using lanthanide (Ln^3+^) ions doped CsPbCl_3_ perovskite nanocrystals (PeNCs). First, K^+^ ions are doped into the lattice to tune the perovskite bandgap by partially substituting Cs^+^ ions, which are well matched to the transition energy of some Ln^3+^ ions from the ground state to the excited state, thereby greatly improving the Förster energy transfer efficiency from excitons to Ln^3+^ ions. Then, creatine phosphate (CP), a phospholipid widely found in organisms, serves as a tightly binding surface-capping multi-functional ligand which regulates the film formation and enhances the optical and electrical properties of PeNC film. Consequently, the Eu^3+^ doped PeNCs based-white LEDs show a peak luminance of 1678 cd m^-2^ and a maximum external quantum efficiency (EQE) of 5.4%, demonstrating excellent performance among existing white PeNC LEDs from a single chip. Furthermore, the method of bandgap modulation and the defect passivation were generalized to other Ln^3+^ ions doped perovskite LEDs and successfully obtained improved electroluminescence (EL). This work demonstrates the comprehensive and universal strategies in the realization of highly efficient and stable white LEDs via single-component Ln^3+^ ions doped PeNCs, which provides an optimal solution for the development of low-cost and simple white perovskite LEDs.

## Abstract (web summary)

Highly efficient and stable white LEDs were fabricated based on Eu^3+^ doped Cs_x_K_1-x_PbCl_3_ PeNCs, in which the PeNC film experienced the band engineering and defect passivation treatment.

## Graphical abstract

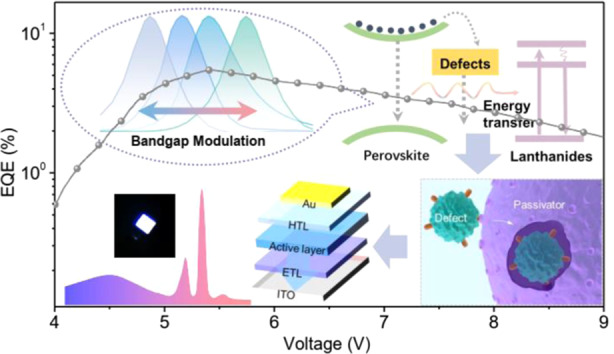

## Introduction

Lead halide perovskite nanocrystals (PeNCs) have gained much attention due to their excellent optoelectronic properties, such as high photoluminescence quantum yield (PLQY), narrow emission line width, emission throughout the visible and near-infrared spectral region (400–950 nm), high exciton binding energy, and composition tunability, which enables them candidates for low-cost solution-processed solid-state lighting source^1–4^. After the rapid progress in the past few years, the metal-halide perovskite light emitting diodes (LEDs) have been developed with external quantum efficiencies (EQEs) of more than 20% for red and green LEDs and more than 12% for blue LEDs^5–7^. Apart from monochromatic light emission, white light emission is also an important indicator of lighting performance^8^. White light perovskite LEDs are possibly to be obtained by stacking different NCs with complementary emissions together in one film^9–11^. However, the halide ion segregation and exchange lead to severe color instability and complex structure in mixed halide perovskite LED devices^12^. Although several attempts have been made, these challenges still limit the generation of stable white light LEDs. Another way for white perovskite LEDs is to produce high PLQY NCs with deep blue emission as an excitation chip. For example, Chen et al. achieved white LEDs with an EQE of 12.2% in which a red PeNC layer was combined with a blue LED^13^. Although this method have relatively mature techniques, there are still some inevitable shortcomings, such as secondary energy consumption, spectral instability, etc.^8^. Therefore, new ideas and technologies are required for the development of white light devices.

Recently, a series of lanthanide (Ln^3+^) ions were successfully incorporated into lattices of the PeNCs and demonstrated expanded and enhanced photonic and electronic properties^14,15^. The Ln^3+^ ions introduce new emitting centers in the perovskite lattice through the energy transfer from excitons to 4*f* or 5*d* levels of Ln^3+^ ions, which is expected to achieve the white light emission in a single-component PeNCs. However, due to the low energy transfer efficiency from the PeNC host to Ln^3+^ ions, as well as the considerably nonradiative relaxation, Ln^3+^ ions contribute little to PL and are difficult to be observed in electroluminescence (EL) devices. Therefore, it is vitally important to improve the energy transfer efficiency from PeNC host to Ln^3+^ ions. Particularly, the successful fabrication of white EL devices from Sm^3+^ doped CsPbCl_3_ NCs with 1.2% EQE in our previous work motivates us to further develop single-component white LEDs^16^, which not only avoid the issues of self-absorption and color instability of other approaches but also simplify the device structure. This kind of single-component white LEDs from a single chip can be considered one of the most ideal white lighting technologies after solving some key issues. The single-component white EL device is realized through the combination of radiative recombination in the PeNC host and the emission of Ln^3+^ ions, as well as the defect emissions generated by Ln^3+^ doping. Firstly, the EL of Ln^3+^ ions originates from the energy transfer process of the perovskite host, which is dominated by the energy matching and the interaction distance between the donor and acceptor. The bandgap of the perovskite host needs to be regulated to match the energy levels of Ln^3+^ ions considering the different 4*f* electronic configurations according to the Förster-Dexter theory^17^. Secondly, it is well known that the surface state of semiconductor NCs has been the dominant factor in determining the optical and electronic properties of the NCs and their applications^18^. Oleic acid (OA) and oleylamine (OAm) are the most widely used surface-capping ligands for the synthesis of PeNCs, showing the poor ability to bind on the NC surface, which is the destabilizing factor for acquiring the stable and efficient EL. Therefore, defect passivation by introducing stronger binding capping ligands is an important approach to improve the performance of PeNCs.

In this work, we present the approaches of energy transfer optimization and defect passivation for fabricating white perovskite LEDs based on Ln^3+^ ions doped all-inorganic PeNCs. First, the energy transfer efficiency between excitons and Ln^3+^ ions is largely improved through the band engineering of doping K^+^ ions to match better with absorption bands of Ln^3+^ ions. Afterward, a small amount of creatine phosphate (CP) is introduced for post-treatment to manage the surface ligand environment of high-quality PeNCs. In organisms, CP acts as an energy reserve replenishing adenosine triphosphate, and appropriate supplements can also improve the body’s immunity. CP is a multi-functional ligand, containing both Lewis-base functional groups (P=O, C=O) and protonic groups (–OH) showing effective defect passivation ability. Similar to the body’s specific immunity, P=O and C=O specifically coordinate with Pb^2+^, –OH groups to form hydrogen bonds with chloride anions, which can both cure the defects of the PeNC surface. Based on an inverse LED device structure, the EL of Ce^3+^, Er^3+^, Sm^3+^, and Eu^3+^ ions doped PeNCs are successfully obtained, respectively.

## Results

**Scheme 1:**
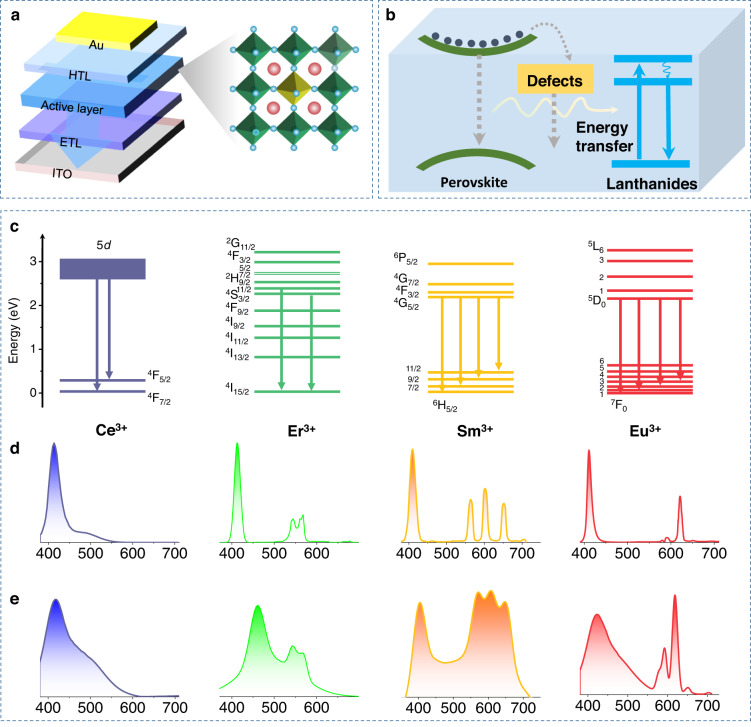
**a** The structure model of Ln^3+^ ions doped CsPbCl_3_ PeNCs. **b** Schematic and energy-level diagrams of the Ln^3+^ ion-doped CsPbCl_3_ LED configuration. **c** Simplified energy-level diagram of Ln^3+^ ion PL process. **d** The PL spectra for the various Ln^3+^ ions doped CsPbCl_3_ PeNCs. **e** The EL spectra for the LEDs based on various Ln^3+^ ions doped CsPbCl_3_ PeNCs

The Ln^3+^ ions doped CsPbCl_3_ PeNCs were synthesized via a hot-injection method, in which the Ln^3+^ ions mainly occupied the sites of Pb^2+^ ions (Scheme 1a). The Ln^3+^ ions doped PeNCs were incorporated into perovskite LEDs as the active layers (based on the device structure of ITO/ZnO/PEI/Perovskite/TCTA (p-type 4,4′,4″-tris(carbazol-9-yl) triphenylamine)/MoO_3_/Au. According to our previous results, the EL of Ln^3+^ ions originates from the resonance energy transfer from excitons to Ln^3+^ ions, which can be described by the well-known Förster-Dexter theory, and the EL is highly dependent on the energy matching between them and the density of trap sites (Scheme 1b). Therefore, we focus on the key issues limiting EL efficiency and investigate such attempts as band engineering and defect passivation. Several Ln^3+^ ions are explored, like Ce^3+^, Sm^3+^, Eu^3+^, and Er^3+^ ions. The PL and EL emission spectra and corresponding transitions are depicted in Scheme 1c–e, in which both the excitonic peaks and the characteristic peaks associated with the intrinsic electronic transitions of Ln^3+^ ions can be observed (5*d*-^4^F_7_/_2_ transition for Ce^3+^, ^4^G_5/2_-^6^H_11/2,9/2,7/2, 5/2_ for Sm^3+^, ^2^H_11/2_/^4^S_/3/2_-^4^I_15/2_ for Er^3+^ and ^5^D_0_-^7^F_J_ (J = 0–4) for Eu^3+^).

**Fig. 1:**
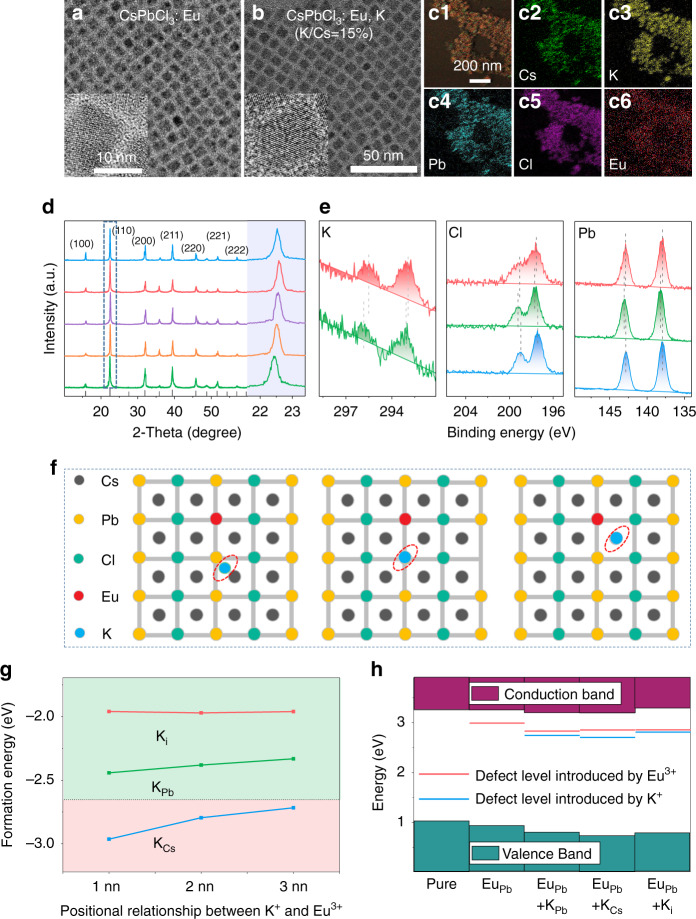
The structure characterization for the Eu^3+^ doped CsPbCl_3_ PeNCs with K^+^ ion co-doping. **a**, **b** TEM images of Eu^3+^ doped CsPbCl_3_ PeNCs without and with 15% K^+^ ion co-doping, respectively. (HTL hole transport layer, ETL electron transport layer) **c** Mapping images of Eu^3+^ doped CsPbCl_3_ PeNCs with 15% K^+^ ion co-doping. **d** XRD patterns of Eu^3+^ doped CsPbCl_3_ PeNCs with various K^+^ ion co-doping concentrations. **e** High-resolution XPS analysis corresponding to K 2*p*, Cl 2*p*, and Pb 4*f* for Eu^3+^ doped CsPbCl_3_ PeNCs without and with 5, 20% K^+^ ion co-doping concentrations, respectively. **f** The diagram of the different positions occupied by K^+^ ions in perovskite. **g** Relative DFEs of Eu^3+^ doped CsPbCl_3_ (dotted line) and Eu^3+^, K^+^ codoped CsPbCl_3_. **h** Defect levels for both Eu^3+^ doped CsPbCl_3_ and Eu^3+^, K^+^ codoped CsPbCl_3_. The red lines represent the defect level introduced by Eu^3+^, whereas the blue lines represent the defect level introduced by K^+^

First, band engineering was carried out with Eu^3+^ ions doped CsPbCl_3_ PeNCs as the typical example. As can be seen from the 4*f* levels diagram, the ^5^L_6_ level locates at about 3.1 eV in energy above the ^7^F_0_ ground state of Eu^3+^ (394 nm), while the exciton emission of CsPbCl_3_ PeNCs situates at about 3.0 eV (410 nm), leading to the mismatch of energy transfer from exciton to the ^7^F_0_-^5^L_6_ transition of Eu^3+^. It is known that the doping of ions can possibly tune the lattice distance and enlarge the bandgap due to the decrease of lattice constant^19,20^. A small amount of potassium ions were introduced into the perovskite lattice by mixing with the Cs-precursor. Figure 1a, b and S1 exhibit the representative transmission electron microscopy (TEM) images of the PeNCs (Eu/Pb of 10% in starting materials) without/with K^+^ doping (K/Cs ratio from 5 to 20%). All the samples distribute uniformly with cubic phase. The average size of Eu^3+^ doped pure CsPbCl_3_ PeNCs is about 7.2 ± 0.3 nm (Fig. 1a). It is found that the size of the as-prepared particles shows the narrow size distributions (Fig. S2). After introducing 5-15% K^+^ ions, the average size of the PeNCs starts to decrease, which decreases to 6.9 ± 0.1 nm with 15% K^+^ ions (Fig. 1b and S1a, b). To further reveal the lattice change, the (110) plane lattice spacing are measured, which decreases from 0.398 nm of PeNCs without K^+^ ions to 0.389 nm of PeNCs with 15% K^+^ ion doping. (Inset of Fig. 1a, b). These results indicate that incorporating K^+^ ions cause the lattice contraction of PeNCs, which may be caused by the partial substitution of some cations in the lattice by K^+^ ions. Further increasing the K^+^ ion doping concentration to 20%, the morphology of PeNCs starts to get messed up, and some fused crystals are formed (Fig. S1c). The actual doping concentrations of Eu^3+^ ions and K^+^ ions determined by ICP-MS are summarized in Table S1. It can be concluded that the actual doping concentrations for K^+^ ions are about 50–60% of the original materials, and is almost independent of the Eu^3+^ doping. The actual concentrations for Eu^3+^, vary significantly with its starting materials. For instance, for Eu/Pb = 5% sample, the actual doping concentration of Eu^3+^ is about 0.7–0.8%. For Eu/Pb = 10% sample, the concentration is 3.5–3.7%. For Eu/Pb = 15% sample, the concentration is 6.5–7.0%. The overdose is important for the sufficient doping of Ln^3+^ ions. The spatial distributions of Cs, Pb, Cl, Eu, and K species in Eu^3+^ ions doped Cs_x_K_1-x_PbCl_3_ (Eu/Pb = 10%, K/Cs = 15%) were further revealed by elemental mapping (Fig. 1c). The homogeneous distribution of the elements among all of the PeNCs suggests the successful introduction of Eu^3+^ ions and K^+^ ions in the perovskite lattice. Then X-ray diffraction (XRD) patterns (Fig. 1d) show that both samples show cubic crystalline structures (refer to PDF no. 75-0408) and no impurity phases formed. Notably, with increasing K^+^ ion doping concentration from 0–15%, the (110) diffraction peaks gradually shift toward the large-angle side, while when the concentration reaches 20%, that diffraction peak shifts slightly toward the small-angle side. The shift to the larger-angle side is consistent with the lattice contraction, which may be caused by the replacement of Cs^+^ (0.167 nm) ions by K^+^ ions (0.138 nm). We deduce that as the K^+^ ion concentrations further increases, K^+^ ions can replace Pb^2+^ ions (0.119 nm) or occupy the interstitial sites, resulting in lattice expansion. These conjectures were verified by X-ray photoelectron spectra (XPS) analysis and density functional theory (DFT). Further increasing the doping concentration of K^+^ ions, the PeNCs easily aggregate to form many large fused crystals (Fig. S3), which can be attributed to the strong bonding of K^+^ ions with chloride ions to form KCl^21^.

For the purpose of identifying the occupying sites of K^+^ ions in Eu^3+^ doped CsPbCl_3_ PeNCs, we performed an XPS test on the Eu^3+^ doped CsPbCl_3_ PeNCs and with 5, 20% K^+^ ion doping, respectively. As provided in Fig. 1e and S4, both the signal of Cs^+^, Pb^2+^, Cl^−^, and Eu^3+^ can be detected in all as-prepared samples. And the signal of K^+^ was detected in K^+^ ions doped samples, which further proved the successful introduction of K^+^ ions. For the sample with 5% K^+^ ion doping, it can be seen that the binding energy (E_b_) of Pb 4*f* shifts to larger E_b_ and that of Cs 3*d* changes little, these shifts in E_b_ are clear evidence for the changes in chemical bonding properties between Pb cations and Cl anions. While the binding energy of Cl 2*p* shifts toward larger E_b_ with 5% K^+^ ion doping, indicating the formation of stronger K-Cl bonding. We speculate that the K^+^ ions tend to occupy the A-sites (Cs^+^) of the perovskite at the low doping concentration of K^+^, which leads to the contraction of PeNC cubic volume, associating with the difference between the ionic radius of Cs (0.167 nm) and K (0.138 nm)^22^. For the sample doping with 20% K^+^ ions, a shift of K 2*p* toward lower E_b_ was observed compared to the PeNCs with 5% K^+^ ions. When the K^+^ ion doping concentration is high, some K^+^ ions enter the interstitial region of the PeNC lattice, which leads to the decrease in the E_b_ of K 2*p*^21^. Meanwhile, the binding energy of Pb 4*f* and Cl 2p shift toward the smaller E_b_ side with 20% K^+^ ions, which could contribute to the expansion of PbCl_6_ octahedral volume, indicating the K^+^ ions partially occupy the Pb^2+^ sites or enter the interstitial region of PeNC lattice. The lattice expansion can also be verified by the increasing (110) plane lattice spacing (inset of Fig. S1c). Therefore, we suggest that at 5% K^+^ ion doping concentration, K^+^ ions mainly occupy Cs^+^ sites, and increasing the concentrations of K^+^ ions will lead to part of K^+^ ions substituting Pb sites or filling the interstitial.

DFT calculations were performed aiming at understanding the changes in structural and photophysical properties of CsPbCl_3_ PeNCs caused by the co-doping of K^+^ and Eu^3+^ ions in the lattice (Supplementary Note 1). According to previous studies^14^, Eu^3+^ ions are set to occupy the Pb^2+^ sites in CsPbCl_3_ PeNCs. A supercell using a (3 × 3 × 3) bulk CsPbCl_3_ unit cell, in which one Pb^2+^ ion is replaced by a Eu^3+^ ion, was used to simulate the doping structure. As shown in Fig. 1f, for the doping of K atom, three different sites were considered, namely interstitial site (K_i_), Pb-substantial site (K_Pb_), and Cs-substantial site (K_Cs_), respectively. We did not consider the condition of K atoms replacing Cl atoms, because the substitution of cations for anions would cause ions to repel each other, leading to an increase in the formation energy of K doping^23,24^. The [PbCl_6_] octahedra exhibits modest distortions around the Eu atom (Fig. S5). While incorporating the K atom to occupy the Pb site or interstitial site, the lattice displays significant distortions near the K-Cl ion pair (Fig. S6a, b). On the contrary, the K atom occupying Cs sites has a certain role in stabilizing the lattice (Fig. S6c). These also can be obtained by calculation of the relative defect formation energies (DFEs). Figure 1g shows the DFE for the doped structures with different charged states of the dopants. The most stable case is that the dopant K atoms exist in the form of occupying Cs atoms, which is more stable than the single doping of Eu atoms. Therefore, combined with our experimental results, it is speculated that in the case of low K^+^ ion doping concentration, the Cs sites are preferentially to be occupied, and the lattice is stabilized to a certain extent. Figure 1h show the calculated bandgaps and the dopant-introduced defect levels of the CsPbCl_3_ perovskite. Obviously, both the Eu^3+^ and K^+^ ions will introduce shallow levels. As is recognized, deep defects can act as carrier traps, leading to nonradiative recombination, whereas shallow defects largely preserve bulk electronic band structure without degrading optoelectronic performance^25^. On the basis of the calculations of the band structure, we can see that the bandgap only shows a small change, in which the change in valence-band maximum (VBM) is more obvious, and the defect levels appear near the bottom of the conduction band. The density of states (DOS) calculation was used to reveal the change in the bandgap. As shown in Fig. S7, the VBM is mainly comprised of Cl orbitals with a negligible Pb component, while the conduction-band minimum (CBM) is formed primarily from Pb orbitals. In order to verify the obvious changes in VBM, we characterize the DOS of the Cl and Pb atoms around K atoms. As shown in Fig. S8, K bond with Cl, and their *p* orbitals overlaps, resulting in a change in the *p* orbital of the Cl atom, which leads to a significant change in VBM (Fig. 1h). The above theoretical results demonstrate that K^+^ ions mainly occupy A sites in Eu^3+^ doped CsPbCl_3_ PeNC system, and emphasize that the structural benefit of K^+^ ion doping is to increase the DFEs, thereby improving the crystal quality of CsPbCl_3_ PeNC host.

**Fig. 2:**
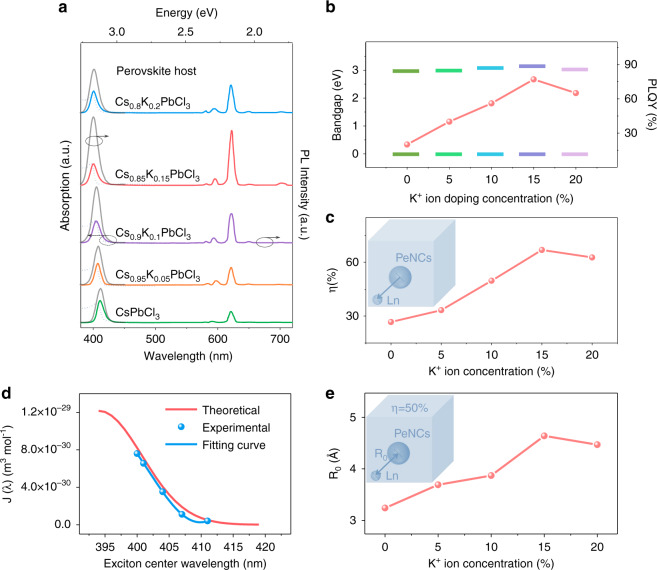
The optical characterization for the Eu^3+^ doped CsPbCl_3_ PeNCs with K^+^ ion co-doping. **a** Absorption spectra (dashed line) /emission spectra (color solid line) of Eu^3+^ doped CsPbCl_3_ PeNCs with various K^+^ ion doping concentrations and the emission spectra (gray solid line) of CsPbCl_3_ PeNCs with various K^+^ ion doping concentrations. **b**, **c** Bandgap of the PeNCs and the corresponding PLQY, energy transfer efficiency of Eu^3+^ doped CsPbCl_3_ with increasing the K^+^ ion-concentration. **d** The dependence curves of the overlap integral *J*(*λ*) and exciton center wavelength *λ* in theoretical and experimental, respectively. **e** Critical binding distance (*R*_*0*_) of Eu^3+^ doped CsPbCl_3_ with increasing the K^+^ ion-concentration

Figure 2a shows the UV-vis absorption (dashed line) and PL emission spectra (solid line) of Eu^3+^ doped CsPbCl_3_ PeNCs with various K^+^ ion doping concentrations. With increasing K^+^ ion doping concentrations, the UV-vis absorption edge gradually shifts toward blue and the bandgap gradually increases from 2.95 to 3.07 eV. This is because K^+^ ions mainly occupy the Cs^+^ sites at the low K^+^ ion concentration, causing the lattice contraction. When the doping concentration exceeds 15%, the absorption edge slightly shifts toward red, about 0.04 eV at 20% K^+^ ion concentration. This is consistent with the fact that some K^+^ ions possibly enter the perovskite interval or replace the Pb^2+^ sites, resulting the lattice expansion. As is known, the lattice shrinkage and size confinement effect may both lead to the blue-shift of the bandgap. In order to evaluate these two effects, we calculated the variation of bandgap based on the equation of quantum confinement effect^26,27^,

1 $$E = \frac{{\hbar ^2\pi ^2}}{{2m_rR^2}} - \frac{{1.786{{{\mathrm{e}}}}^2}}{{4\pi \varepsilon _0\varepsilon R}}$$

where *R* is the particle radius, *m*_*r*_ is the effective mass of the exciton, and *ε* is the relative dielectric constant of CsPbCl_3_ bulk material. The result shows that the bandgap merely shifts about 6 meV (Table S2) as the particle size changes from 7.2 to 6.9 nm, which is much smaller than the practical change (2.95 to 3.07 eV). Thus we can conclude that the variation of the bandgap is mainly caused by lattice shrinkage, rather than the quantum confinement effect. The increase of bandgaps of PeNC host results in a better matching between the excitonic emission band and absorption band of Eu^3+^ ions^17,28^, which can promote more efficient energy transfer from the PeNC host to Eu^3+^ ions. It can be seen that with the introduction of K^+^ ions, the emission peak of the perovskite host shifts from 411 to 400 nm (gray solid line in Fig. 2a). The intensity of the exciton emission decreases and the intensity of red emission originated from the Eu^3+^ ions increases significantly with Eu^3+^ ions introduced, which proves the energy transfer from perovskite host to Eu^3+^ ions. The PLQYs were measured to further evaluate the optical performances for the Eu^3+^ and K^+^ ions codoped PeNCs (Fig. 2b and Table S3). As a function of K^+^ concentration, the PLQY for the emissions of exciton and Eu^3+^ ions initially increases, approaches an optimum, and then decreases with the increase of the K^+^ ion doping concentrations. The optimal PLQY reaches 77% at 15% K^+^ ion doping concentration. The introduction of K^+^ ions can effectively increase the bandgap of the CsPbCl_3_ PeNC host, which benefits the energy transfer from perovskite to Eu^3+^ ions (Fig. 2b). In order to further investigate the effect of the K^+^ ions on the energy transfer efficiency from PeNC host to Eu^3+^ ions, the luminescent dynamic processes were measured. The dynamics recorded at 400 nm for the *x*% K^+^ doped CsPbCl_3_ (x = 0–20) PeNCs were presented in Fig. S9. The PL decay curves were fitted with a multi-exponential function to determine the PL decay lifetimes, and the calculated radiative decay rates (*k*_*r*_), the nonradiative decay rates (*k*_*nr*_) (details in Supplementary Note 2) were summarized in Table S4. The average lifetime of the excitonic emission increases obviously with increasing the K^+^ ion concentrations, and the radiative decay rate increases about twofold, the nonradiative decay rate decreases about 12-fold in Cs_0.85_K_0.15_PbCl_3_ PNCs, signifying that the *x*% K^+^ doped CsPbCl_3_ possesses fewer trap states^29^. After the incorporation of Eu^3+^ ions, the excitonic emission decays faster due to the energy transfer from the perovskite host to the Ln^3+^ ions (Fig. S10). The energy transfer efficiency (*η*) from perovskite host to Eu^3+^ ions can be calculated by the equation^30^

2 $$\eta = 1 - \frac{{\tau _{Eu}}}{{\tau _0}}$$

where *τ*_*0*_ and *τ*_*Eu*_ are the exciton relaxation time constant of *x*% K^+^ doped CsPbCl_3_ and *x*% K^+^- 10% Eu^3+^ codoped CsPbCl_3_ PeNCs, respectively. As shown in Fig. 2c, the energy transfer efficiency gradually increases with increasing K^+^ doping concentrations, and the optimum efficiency approaches 66.9%. When the wavelength at 622 nm is monitored, the decay time constant varies from 0.614 to 0.904 ms with increasing K^+^ doping concentration (Fig. S11), which again indicates these emission peaks originated from Eu^3+^ ions. In order to distinguish whether the energy transfer from PeNC host to Eu^3+^ ions is a radiative or nonradiative process, the PL lifetime for the samples at fixed K^+^ ion concentration decreases with increasing Eu^3+^ ion concentrations (Fig. S12). The energy transfer efficiency (Table S5) gradually increases with increasing Eu^3+^ doping concentrations, and the optimum efficiency approaches 75% at 6.5 mol% Eu^3+^ concentration. And 1/η displays a linear relationship with R^6^ (Fig. S13), indicating that the interaction between Cs_0.85_K_0.15_PbCl_3_ and Eu^3+^ ions should be a Förster energy transfer process.

Moreover, according to the Förster energy transfer theory^28,31^: *η*∝*R*_*0*_∝*J*(*λ*), where *η* is the energy transfer efficiency, *R*_*0*_ is the critical distance, and *J*(*λ*) is the overlap integral of the emission spectrum of the donor (PeNC host) and the absorption spectrum of the acceptor (Eu^3+^ ions). The spectral overlap is calculated as:

3 $${{{\mathrm{J}}}}(\lambda ) = {\int}_0^\infty {F_D\left( \lambda \right)\varepsilon _A\left( \lambda \right)\lambda ^4{{{\mathrm{d}}}}\lambda }$$

where *F*_*D*_(*λ*) is the emission of the PeNC host with an integrated intensity normalized to unity, and *ε*_*A*_(*λ*) is the molar extinction coefficient of the Eu^3+^ ions at wavelength *λ*. Here, we use this formula to simulate the relationship between *J*(*λ*) and central emission wavelength of PeNC host. We set the PL of the PeNC host and the absorption band of Eu^3+^ ions as a Gaussian curve (detail was shown in Supplementary Note 3). The simulated *λ*-*J*(*λ*) curve is shown in Fig. 2d (red line), from which we can see that the spectral overlap *J*(*λ*) increases with the blue-shift of the host emission center. Subsequently, the actual spectral overlap of PL emission of Cs_1-x_K_x_PbCl_3_ and UV-vis absorption of Eu^3+^ ions was characterized (Fig. S14 and blue line in Fig. 2d), which is in good agreement with the theoretical simulation curve. This also demonstrates the importance of bandgap regulation in obtaining efficient energy transfer from perovskite to Ln^3+^ ions. And the critical distance *R*_*0*_ can be calculated by the formula:

4 $$R_0^6 = \frac{{9000\left( {\ln 10} \right)k^2Q}}{{128\pi ^5N_An_r^4}}J\left( \lambda \right)$$

where *k*^2^ is the spatial orientation factor of the dipole, *Q* is the quantum yield of the PeNCs in the absence of Eu^3+^ ions, *N*_*A*_ is Avogadro’s number, *n*_*r*_ is the refractive index of the medium. At critical distance *R*_*0*_, the energy transfer efficiency is equal to the radiative decay rate. The calculated results were shown in Fig. 2e, the *R*_*0*_ varies from 3.24 to 4.64 Å with increasing K^+^ doping concentrations, indicating that the introduction of K^+^ ions increases the R_0_, thus increasing the energy transfer efficiency from the PeNC host to Eu^3+^ ions.

**Fig. 3:**
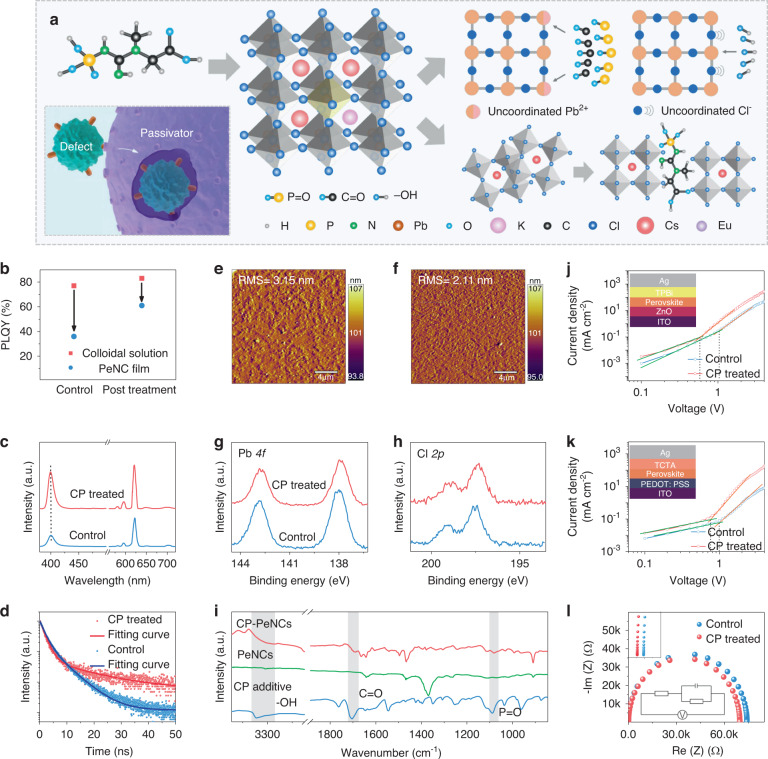
Interactions between PeNCs and CP passivator. **a** Schematic diagram of CP-passivated PeNCs. **b** PLQYs of PeNC colloidal solution and corresponding film without and with CP treated. **c**, **d** PL spectra and PL decay curves of PeNC film without and with CP treated. **e**, **f** AFM images of of PeNC film without and with CP treated. **g**, **h** High-resolution XPS analysis corresponding to Cl 2*p*, Pb 4*f* without and with CP treated, respectively. **i** FTIR spectra of pure CP and control, CP-treated films. **j**, **k** The electron-only devices and hole-only devices of control and CP-treated PeNCs. The inset at the top gives the device structure. **l** Cole-Cole plots of LEDs based on the pristine and modified PeNCs. The inset at the top left shows the enlarged Cole-Cole plots in the range of 0–100 Ω. The inset at the bottom gives the equivalent circuit of the devices

Although the K^+^, Eu^3+^ codoped colloidal solution shows a very high PLQY, it still exhibits a partial decrease of PLQY in the film due to the aggregation of particles and the existence of surface defect states^32–34^. Therefore, we pursue clearing the uncoordinated sites on the NC surface to improve the film performance. We learned that the immune cells can clear the pathogens through the specific binding of proteins (inset of Fig. 3a). Inspired by this, we select the CP, a phospholipid widely found in organisms, which contains both Lewis-base functional groups (C=O and P=O) and protonic groups (–OH) (Fig. 3a). The C=O and P=O bonds can specifically bind the uncoordinated Pb sites on the surface of PeNCs, which works like immune cells clearing specific pathogens to passivate the defect of PeNC film. The tighten binding of CP ligand on the surface of PeNCs could reduce the aggregation of particles. The CP additive was introduced via a post-treatment method. As can be seen from Fig. 3b, the PLQY of the control PeNC film is much lower than that of the corresponding colloidal solution. For the samples treated with CP, the PLQY of the solution is increased to 83%, and the PLQY of the corresponding film is greatly increased to 61% as well. The PL stability of the CsPbCl_3_: Eu^3+^ PeNC film, CsPbCl_3_: K^+^, Eu^3+^ PeNC film, and CP-treated K^+^, Eu^3+^ codoped CsPbCl_3_ PeNC film was compared under atmospheric conditions. Whereas it took 84 days for CP-treated K^+^, Eu^3+^codoped CsPbCl_3_ PeNC film to lose 70% of its PL intensity (Fig. S15). We believe that the better stability of the CP-treated device is due to its high crystallinity with less defects^35,36^. Subsequently, the PL spectra were collected to identify the effect of CP on the optical properties of PeNC films (Fig. 3c). Both samples show a similar excitonic emission peak around 400 nm. A slightly blue-shift of excitonic PL peak in the treated film was observed, which can be ascribed to the passivation effect on the defects that locate above or below the VBM or CBM of PeNCs. A similar PL blue-shift was also reported earlier for the Lewis-base passivated defects of perovskite^37,38^. The defect passivation is also evident from the prolonged lifetime of excitonic emission (Fig. 3d)^39^, where the control film initially exhibits the average lifetime 〈*τ*〉 = 3.6 ns, which increases to 〈*τ*〉 = 5.6 ns after CP treatment. Compared with the control samples, CP-treated samples show little change in the average size (Fig. S16). We also confirm that the treatments do not measurably change the crystal structure of the PeNC as evidenced by XRD (Fig. S17), and the (110) crystal plane of CP-treated film shows narrower full-width at half maximum, indicating better crystallization^40–42^. Scanning electron microscope (SEM) characterization indicates that CP-treated PeNC films display improved film morphology compared with the control sample (Fig. S18). As shown in Fig. 3e, f, atomic force microscope (AFM) characterization was used to further check the morphology. The control film shows the root-mean-square (RMS) roughness of 3.15 nm. For the CP-treated sample, the RMS value of the film reduces to 2.11 nm, confirming that a smooth nanocrystalline perovskite film with less particle aggregation is formed. Then XPS and Fourier-transform infrared spectroscopy (FTIR) were performed to investigate the interaction between CP and PeNCs. Compared with the control film, CP-treated film shows core-level peaks of Pb 4*f* and Cl 2*p* that shift towards lower E_b_ (Fig. 3g, h). The shifts of Pb 4*f* could be ascribed to that the C=O and P=O moiety can donate their lone electron pairs on the oxygen atoms to the empty 6*p* orbital of Pb^2+^. This also leads to an increase in the electrostatic interaction between the Pb^2+^ and the Cl^-^ ions and thereby reduces the E_b_ of Cl 2*p*^43^. As verified via FTIR spectra (Fig. 3i), the pure CP exhibits a peak from C=O stretching vibrations at 1705 cm^−1^, which shifts to 1692 cm^−1^ in the CP-treated film, while the P=O stretching vibration peak at 1088 cm^−1^ moves to 1078 cm^−1^^44,45^. We speculate that new bindings between C=O, P=O bonds and PeNCs are formed. Considering the XPS results, we think the C=O and P=O bonds tend to specifically bind with uncoordinated Pb^2+^ ions and repair the surface defects. The –OH vibration signal of the CP additive becomes broader and weaker in the CP-treated PeNCs, indicating the formation of hydrogen bonds between the -OH functional group and the chloride anion^46^, which can promote the quality of the PeNC film through the center of nucleation negatively charged defects via hydrogen bonding.

To better understand the role of this post-treatment, the single carrier (electron-only and hole-only) transported devices for the pristine and modified PeNCs were fabricated to analyze quantitatively the trap state densities and the carrier mobility of different PeNC films via the space-charge-limited-current region (SCLC) method (Fig. 3j, k and Fig. S19). The defect density is calculated according to the equation^47,48^:

5 $${{{\mathrm{N}}}}_{{{\mathrm{t}}}} = \frac{{2\varepsilon \varepsilon _0V_{TFL}}}{{eL^2}}$$

where *ε* and *ε*_*0*_ are the relative dielectric constant of the PeNCs and the vacuum permittivity, respectively, *V*_*TFL*_ is the trap-filled limit voltage, *L* is the thickness of the obtained PeNC film, and *e* is the elementary electronic charge. The trap state densities in the modified films (3 mg L^−1^ CP) are *N*_*t*_(*e*) = 2.7 × 10^16^ cm^−3^ and *N*_*t*_(*h*) = 3.5 × 10^16^ cm^−3^ (where the subscripts “e” and “h” represent electrons and holes, respectively), which are approximately half those of the pristine sample (Table S6). The fall-off in trap state densities indicates the successful passivation of surface defects via the ligand treatment with CP. The well-passivated PeNCs can sufficiently suppress the nonradiative recombination caused by the surface defects, agreeing with the results of lifetime measurement. We also obtain the carrier mobilities of the PeNC films according to the Mott–Gurney law^47^:

6 $${{{\mathrm{J}}}} = \frac{{9\varepsilon \varepsilon _0\mu V^2}}{{8L^3}}$$

where *ε*_*0*_ is the vacuum permittivity, *ε* is the average relative dielectric constant of CsPbCl_3_ (*ε* ≈ 4.432), *L* is the thickness of the PeNC film, and *J*, *μ*, and *V* are the measured current density, carrier mobility, and applied voltage, respectively. The calculated electron mobility and hole mobility are 3.1 × 10^-4^ and 2.1 × 10^-4^ cm^2^ V^−1^ s^−1^ for the pristine PeNCs (Table S7), and 4.1 × 10^−4^ and 3.8 × 10^-4^ cm^2^ V^−1^ s^−1^ for the modified PeNCs, respectively. Obviously, the electron and hole mobilities are much closer for the modified PeNC films, benefiting from the balance injection in the LEDs^49^. In fact, charge mobilities can be increased by defect passivation to suppress the charge scattering, which will increase the charge injection efficiency and promote the charge balance in the LED devices^50,51^. In addition, electrochemical impedance spectroscopy (EIS) was used to further characterize the effect of additive on the electrical properties of the LEDs, the equivalent circuit was shown in the inset in Fig. 3l. As previously reported^52^, the charge transfer resistance (*Rs*) at a lower frequency range reflects the charge transfer resistance at the interface between the charge transport layer and the active layer, and the carrier recombination resistance (*R*) at a higher frequency range is related to the carrier recombination in the active layer. According to our experimental results, the *Rs* values are 47 and 26 Ω for the LEDs without and with CP treated, respectively. At the same time, the *R* value decreases from 74,232 Ω for the pristine LEDs to 69,763 Ω for the CP-treated LEDs. These results indicate that the recombination of carriers in the active layer was ameliorated by balancing the injection of carriers in the LEDs.

**Fig. 4:**
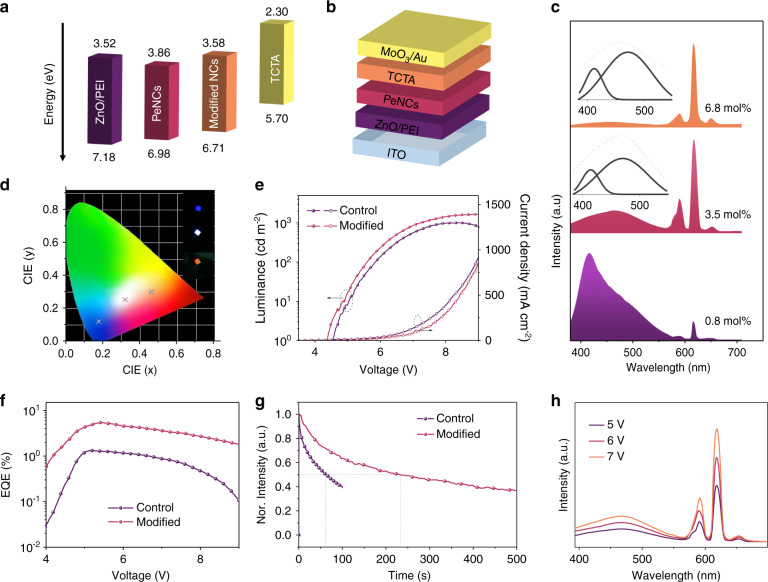
Optoelectronic characteristics of the PeLEDs based on Eu^3+^, K^+^ ion codoped CsPbCl_3_ PeNCs. **a** Schematic of the modified Eu^3+^, K^+^ ion codoped CsPbCl_3_ LED configuration. **b** Energy-level diagrams for each functional layer in the LEDs. **c** EL spectra and **d** CIE coordinates for the LEDs based on the PeNCs with different Eu^3+^ ion doping concentrations. Inserts of **d**: Photographs of LEDs with different Eu^3+^ ion doping concentrations. **e** Current densities and luminescence, **f** EQE-V curves of LEDs based on 3.5 mol% Eu^3+^ ion doping concentration in PeNCs without and with CP modified. **g** Operational stability lifetime of LEDs based on 3.5 mol% Eu^3+^ ion doping concentration in PeNCs without and with CP modified. **h** EL spectra against voltage of white LEDs

Based on the optical and electrical properties of the modified K^+^, Eu^3+^ codoped PeNC film, the fabrication of high-performance LEDs is promising. As shown in Fig. 4a, the device structure is ITO/ZnO/PEI/Eu^3+^ doped Cs_0.85_K_0.15_PbCl_3_ PeNCs/TCTA /MoO_3_/Au (details are shown in the Materials and methods Section). The thicknesses of ITO, ZnO/PEI, PeNCs, TCTA, and MoO_3_/Au were 100, 40, 30, 50, and 40 nm, respectively (Fig. S20). The energy diagrams of LEDs (Fig. 4b) reveal that both types of devices can achieve barrier-free injection. The energy levels of the pristine and CP-modified PeNC films were explored by ultraviolet photoelectron spectroscopy (UPS) measurement (Fig. S21). It can be found that after CP modification, the bandgap has no obvious change, and the VBM is slightly increased, which may be caused by the change of the surface chemistry^53,54^, thereby reducing the hole injection barrier and improving the charge carrier injection balance. The normalized EL spectra of the LEDs based on PeNCs with concentrations of 0.8, 3.5, and 6.8 mol% Eu^3+^ doping concentration are depicted in Fig. 4c. We also found the inhomogeneous broadening of excitonic peaks in EL spectra relative to that in PL spectra. In order to figure out the variation from EL to PL, the excitonic peaks were decomposed in the EL spectra with a Gaussian function. As shown by the dashed line in Fig. 4c, the existence of a broadening excitonic peak and a broad-fitted component centering at 470 nm spanning from 400 to 800 nm can be observed in each resolved spectral line. The red-shift and broadening of an excitonic peak in the EL spectra in comparison to the PL spectra was observed (Fig. S22). The red-shift may be caused by the dielectric environment of the surrounding medium, the energy transfer from small quantum dots to larger quantum dots in the ensemble film, and the spectral broadening is attributable to that a larger exciton polarization under the electric field, resulting in the increased LO (longitudinal optical)-phonon coupling^55–58^. In addition, we suggest that the broad-fitted component centering at 470 nm may be associated with Eu^3+^ induced energy levels as the broad EL is absent in LEDs based on pure CsPbCl_3_ and Cs_0.85_K_0.15_PbCl_3_ NCs (Fig. S23a, b). For the LED device based on Eu^3+^ ion-doped CsPbCl_3_ NCs, similar broad EL is also observed (Fig. S23c). As we discussed in DFT calculations, the Eu^3+^ ions introduce an energy level below the CBM, which suits well with the broad component of EL. Under the excitation of an electric field, carriers can easily fill on the defect levels, the similar behavior has been studied in the LEDs based on nanocrystalline semiconductors or carbon dots^59,60^. The Commissions Internationale de l’Eclairage (CIE) chromaticity coordinates of the as-prepared LEDs are depicted in Fig. 4d. Increasing the concentrations of Eu^3+^ ions from 0.8 to 6.8 mol% enables us to achieve tunable CIE coordinates of LEDs from blue to orange regions, which originates from the excitonic band and Ln^3+^ ions, as well as the emitting component of defects. Importantly, based on the PeNCs with the Eu^3+^ ion doping concentration of 3.5 mol%, a single-component white light EL with the CIE of (0.32, 0.25) is successfully obtained, which avoids the problem of halide ion exchange for multi-component white light emitting perovskite LEDs.

Then we discuss the role of the passivate agent in the performance improvement of white perovskite LEDs. Figure 4e shows the current density-voltage-light intensity (*J*-*V*-*L*) curves of white LEDs with and without CP. The perovskite LEDs with CP passivation exhibit stronger luminance than that of the pristine PeNC counterpart in the whole voltage range and reach the maximum luminance of 1678 cd m^−2^, which is much higher than 1008 cd m^−2^ for LEDs based on pristine PeNCs. Simultaneously, the best-performance device without CP passivation shows an EQE of 1.3%, while the CP-treated device shows EQE as high as 5.4% (Fig. 4f). We attribute the improved device performance to the superior PLQY and enhanced carrier balance in the modified PeNCs. We then measured the device operating stability of white perovskite LEDs, applying a constant voltage of 6 V, in which the half-lifetime (T_50_) was measured to be 220 s, twice that in control devices (Fig. 4g and Supplementary Video S1). The increased operating stability benefits from the ligand-induced carrier balance, while K^+^ ions have little effect on the stability (Fig. S24). In comparison with the recent works on white light perovskite LEDs (Table S8), the as-prepared perovskite LED displays a high EQE and relatively high brightness, which is a competitive advantage for practical application. The EL spectra under different bias voltages were investigated, as shown in Fig. 4h, the shape of the EL spectra almost keep unchanged, benefiting from the as-developed single-component white light LED device, in which the CIE coordinate values fluctuate little, from (0.32, 0.25) to (0.33, 0.25) (Fig. S25). The EQE histogram for 30 devices based on control, CP-treated PeNCs are presented in Fig. S26, indicating that our devices show good reproducibility. Overall, we have provided a novel approach for achieving efficient and stable single-component white light emitting LEDs.

**Fig. 5:**
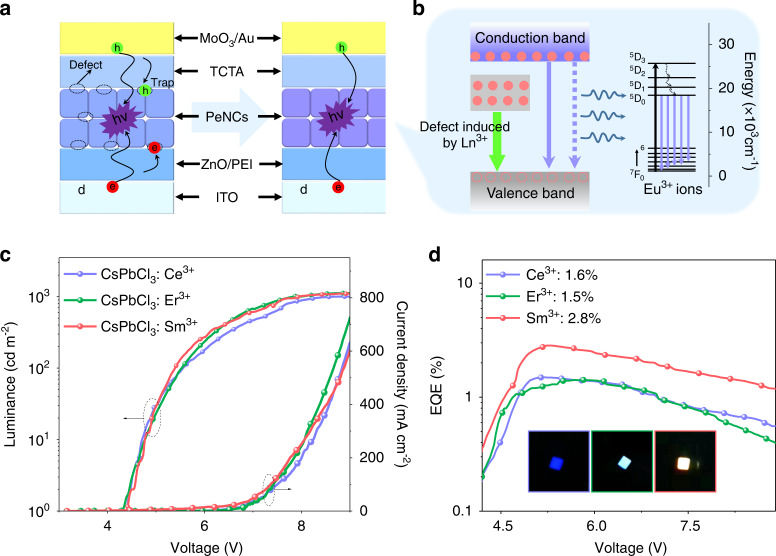
Perocskite LEDs based on Ln^3+^ ion-doped CsPbCl_3_ PeNCs. **a** Structure of LED based on PeNCs without and with passivation. Schematic illustrates that CP could passivate defects, in which the defects may trap carriers (e.g., holes and electrons), decrease exciton recombination, and hence degrade the device performances. **b** The possible EL mechanisms for the LEDs based on Ln^3+^ doped PeNCs. **c** Current density and luminance, **d** EQE-*V* curves of LEDs based on Ln^3+^ ion-doped CsPbCl_3_ PeNCs

The strategy based on the K^+^ ion modulated bandgap was further used to improve energy transfer from the perovskite host to Ce^3+^ and Sm^3+^ ions. With the introduction of K^+^ ions, the emission peak of the perovskite host occurs blue-shift (Fig. S27), which matches better for the ^2^H_5/2_-^6^P_5/2_ and ^4^F_7/2_-5*d* transitions of Sm^3+^ and Ce^3+^, respectively. For some other Ln^3+^ ions, the bandgap needs to be reduced to fit with the energy levels of Ln^3+^ ions and improve the energy transfer efficiency. We have used the anion exchange method to match the bandgap with the ^4^I_15/2_-^4^F_5/2_ transition of Er^3+^ ions. With the bandgap decreasing, the fluorescence ratio of Er^3+^ ions to the PeNC host increases (Fig. S28). Similar to Eu^3+^ ions, the modulation of bandgap increases the energy transfer efficiency of the PeNC host to Ln^3+^ ions (Table S9), which have been calculated by the luminescent dynamic processes (Fig. S29). During the film-forming process, the defects induced by unsaturated sites would capture electrons and holes, thus resulting in low device efficiency. The interaction between uncoordinated Pb and C=O, P=O from CP was deemed to be the key factor that passivate the defects of PeNCs, which could decrease nonradiative recombination, and thus improve the device performances (Fig. 5a). Therefore, on the basis of bandgap modulation, we also performed CP post-treatment on Ln^3+^ doped PeNCs and prepared the LED devices. In the perovskite LEDs based on Ln^3+^ ions doped PeNCs, the carriers created at the electrode are transmitted through the carrier transport layers to the active layer and the defect state (induced by Ln^3+^) under the electric field^59,61^ and subsequently emit photons through radiative recombination, resulting in the EL of PeNC host and defect, respectively. Alternatively, partial excitons in the active layer transfer energy to the upper intrinsic states of Ln^3+^ ions benefiting from the perfect match of the energy gaps between excitonic recombination, and then transmit to the emitting levels (^5^D_0_) through the nonradiative relaxation, such as the case of Eu^3+^ ions (Fig. 5b), thus leading to the EL of Ln^3+^ ions. The EL spectra of the LED devices before (Scheme 1e) and after (Fig. S30) energy band regulation and defect passivation were compared, in which the EL intensity of Ln^3+^ ions enhanced. *J*-*V*-*L* and EQE-current density curves of the LEDs are shown in Fig. 5d, e. The devices show the maximum luminance and EQE of 1120, 875, 1100 cd m^−2^, 1.6, 1.5, and 2.8% for the LEDs based on Ce^3+^, Er^3+^, and Sm^3+^ doped PeNCs, respectively.

## Discussion

In summary, we have developed a stable wide-bandgap A-site mixed Eu^3+^ doped Cs_x_K_1-x_PbCl_3_ PeNCs, and the wide-bandgap PeNC host can ensure efficient energy transfer from exciton to the Ln^3+^ ions. To overcome the efficiency loss of spin-coating thin films from colloidal solution, we passivated the PeNCs with CP molecules that offer strong binding with the surface Pb^2+^ cations and Cl^−^ anions, from which the optimum PLQY approaches 83%. Efficient and spectra-stable LEDs were fabricated based on the active layers of modified PeNCs with various Eu^3+^ doping concentrations. The as-prepared LEDs demonstrated tunable EL from blue to orange, contributed by the efficient energy transfer from the CsPbCl_3_ perovskite host to Eu^3+^ ions. Most importantly, the single-component white light emitting LEDs were realized based on Eu^3+^ (3.5 mol%) doped K_0.15_Cs_0.85_PbCl_3_ PeNCs, which shows the peak EQE of 5.4%, demonstrating quite excellent performance in white single-component perovskite-based LEDs up to now. Furthermore, LED devices based on Ce ^3+^, Er^3+^, and Sm^3+^ ions doped PeNCs were achieved with the bandgap optimization and defect passivation method. Our results indicate that the broad component of EL originated from the defects induced by Ln^3+^ ions. This work demonstrates the superiority of single-component Ln^3+^ ions doped perovskite in the realization of highly efficient and stable white LEDs, providing the optimal scheme for the development of low-cost, simple-structure white perovskite LEDs.

## Materials and methods

### Materials

Cesium acetate (CsOAc, 99.9%), 1-octadecene (ODE, 90%), oleic acid (OA, 90%), oleylamine (OAm, 70%), lead chloride (PbCl_2_, 99.99%), europium acetate hydrate (Eu(OAc)_3_^.^3H_2_O, 99.99%), cerium acetate hydrate (Ce(OAc)_3_^.^4H_2_O, 99.99%), erbium acetate hydrate (Er(OAc)_3_^.^4H_2_O, 99.99%), samarium acetate hydrate (Sm(OAc)_3_^.^6H_2_O, 99.99%), potassium acetate (KOAc, 99.9%, ACS grade, Fischer), ethanol (99%), zinc acetate (99.99%), sodium hydroxide (NaOH, 99.99%), toluene (99,95%), were all purchased from Sigma-Aldrich and were used as starting materials without further purification.

### Synthesis of Cs-oleate

0.06 g of CsOAc was loaded into a mixture of 3.75 mL of ODE and 0.35 mL of OA, keep a vacuum, and then heated to 150 °C until all the powder was completely dissolved.

### Synthesis of Ln^3+^ doped CsPbCl_3_ PeNCs

First, Ln(OAc)_3_^.^xH_2_O (0.05, 0.1, 0.15, 0.2, 0.3, 0.35 mmol) was dissolved in 0.5 mL ethanol and then dispersed in the ODE (10 mL) and OA (2 mL) mixed solution. Then, the temperature was elevated to 130 °C under the protection of N_2_ gas for dissolving Ln(OAc)_3_^.^3H_2_O sufficiently. Next, PbCl_2_ (0.3 mmol) and OAm (3 mL) were added at the same time, and the temperature was kept at 130 °C for 1 h. After dissolved completely, the temperature was raised to 220 °C. The as-prepared Cs-oleate (1 mL) was injected into the contents promptly. After 10 s, the reactant was immediately transferred to an ice-water bath. The resulting products were centrifuged at 5000 rpm for 10 min and the supernatant was discarded. The precipitates were dispersed in toluene and then centrifuged at 9500 rpm for 10 min. The resultant PeNCs were dispersed in toluene.

### Preparation of double cation (Cs, K ions) precursor

0.06 g CsOAc and 0.015, 0.03, 0.045, 0.06, and 0.075 mmol potassium acetate was loaded into a mixture of 3.75 mL of ODE and 0.35 mL of OA under N_2_ gas protection and then was heated to 150 °C until all the powder was completely dissolved.

The synthesis process for the Cs_x_K_1-x_PbCl_3_: Eu^3+^ PeNCs was similar to that of Ln^3+^ doped CsPbCl_3_ PeNCs. The only difference was that the double cation precursor was injected in the hot-injection part.

### Bandgap modulation for Ln^3+^ (Ln = Ce, Er, Sm) doped PeNCs

#### Ce^3+^ doped PeNCs

K^+^ ions were incorporated to modulate the bandgap. Ce(OAc)_3_^.^4H_2_O (0.05, 0.1, 0.15, 0.2, 0.3, 0.35 mmol) was dissolved in 0.5 mL ethanol and then dispersed in the ODE (10 mL) and OA (2 mL) mixed solution. And the following steps were the same as the synthesis of the Cs_x_K_1-x_PbCl_3_: Eu^3+^ PeNCs.

#### Er^3+^ doped PeNCs

The Br^-^ source was used for an anion exchange experiment to tune the bandgap. Finally, the CsPbCl_1.5_Br_1.5_ PeNC host was selected for device fabrication.

##### Anion exchange experiment

PbBr_2_ used as Br^-^ sources were mixed with ODE (5 mL) in a 25 mL three-neck flask and kept under vacuum at 120 °C for 10 min. OA and OAm (0.2 mL each) were injected at 120 °C under N_2_ protection. The anion source, after complete solubilization, was added to the NC solution (1 mmol) dropwise at 120 °C. After the reaction for 5 min, NCs were isolated by centrifugation. The supernatant was discarded and the precipitate was dispersed in toluene and centrifuged again.

#### Sm^3+^ doped PeNCs

K^+^ ions were incorporated to modulate the bandgap. Sm(OAc)_3_^.^6H_2_O (0.05, 0.1, 0.15, 0.2, 0.3, 0.35 mmol) was dissolved in 0.5 mL ethanol and then dispersed in the ODE (10 mL) and OA (2 mL) mixed solution. And the following steps were the same as the synthesis of the Cs_x_K_1-x_PbCl_3_: Eu^3+^ PeNCs.

### CP post-treatment

CP additive was dissolved in toluene and stirred overnight. About 500 μL of this solution was added to the obtained PeNC solution. Then, the mixture was centrifuged at 9500 rpm for 10 min, dispersed resultant PeNCs in 2 mL toluene.

### Synthesis of ZnO NCs

First, 0.4403 g zinc acetate and 30 mL ethanol were added into a 100 mL three-neck flask. Then the solution was heated to 30 °C for 30 min after 10 min N_2_ flow. A solution (0.2 g sodium hydroxide dissolved in 10 mL ethanol) was quickly injected, and the mixture was kept stirring for 4 h at room temperature under N_2_. After centrifugation, the obtained products were dissolved in ethanol.

### LED device fabrication

Indium tin oxide (ITO) glass substrates were cleaned by UV-ozone treatment for 15 min. The ETL was prepared via spin-coating ZnO NC solution onto the ITO substrates at 1000 rpm for 40 s and annealed in air at 150 °C for 10 min, and the solution of polyethyleneimine (PEI) (dissolved in 2-methoxyethanol, 0.2% mass ratio) was spin-coated onto the ZnO film at a speed of 3000 rpm and annealed at 125 °C for 10 min. Then, Eu^3+^ doped CsPbCl_3_ PeNCs (20 mg mL^−1^) was deposited by spin-coating at 1000 rpm for 50 s. TCTA, MoO_3_, and Ag were then sequentially deposited by thermal evaporation in a vacuum deposition chamber (1 × 10^−7^ Torr).

### Characterizations

Powder XRD patterns were recorded by using a Rigaku D/Max-Ra X-ray diffractometer with a monochrome at Cu Kα radiation (λ = 1.54178 Å). For TEM measurements, a Titan transmission electron microscope (FEI Company) operated at 300 kV was used. For the HR-TEM measurements, the samples were imaged in EF-TEM mode with a 20 KeV energy slit inserted around the zero energy loss of electrons. Trace Metal Analysis was carried out using inductively coupled plasma optical emission spectrometry (ICP-OES) on a Varian 720-ES ICP-optical emission spectrometer. In the measurements of luminescent dynamics, the samples were pumped using a laser system consisting of an Nd: YAG pumping laser (1064 nm), a third-order Harmonic-Generator (355 nm), and a tunable optical parameter oscillator (OPO, Continuum Precision II 8000) with a pulse duration of 10 ns, a repetition frequency of 10 Hz and a line width of 4–7 cm^−1^. A visible photomultiplier (350–850 nm) combined with a double-grating monochromator was used for spectral collection. UV-Vis absorption spectra were obtained by using a Shimadzu UV-3101PC scanning spectrophotometer. The samples were pumped using a laser system consisting of an Nd: YAG pumping laser (1064 nm), a third-order Harmonic-Generator (355 nm), and a tunable optical parameter oscillator (OPO, Continuum Precision II 8000) with a pulse duration of 10 ns, a repetition frequency of 10 Hz and a line width of 4–7 cm^−1^. A visible photomultiplier (350–850 nm) combined with a double-grating monochromator were used for spectral collection. The PLQYs of the samples were acquired using an integrating sphere incorporated into a spectrofluorometer (FLS980, Edinburgh Instruments). Quantum yield was then calculated by using the Edinburgh L980 software package. X-ray photoelectron spectroscopy (XPS) was carried out in a Kratos Axis Ultra DLD spectrometer equipped with a monochromatic Al Kα X-ray source (hν = 1486.6 eV) operated at 150 W with a multichannel plate, and a delay line detector under 1.0 × 10^−9^ Torr vacuum. The energy levels of PeNC films were measured using an integrated ultrahigh vacuum system equipped with a multi-technique surface analysis system (VG Scienta R3000) with an excitation energy of 21.218 eV and were determined by ultraviolet photoelectron spectroscopy (UPS). The current-voltage characteristics of the devices were measured with a Keithley 2612B source meter and the LED brightness was determined using a Photo Research Spectra Scan spectrometer PR650.

## Supplementary information

supplementary information

Supplementary Video S1

Confidential Review

## References

[1] Zhou DL (2017). Cerium and ytterbium codoped halide perovskite quantum dots: a novel and efficient downconverter for improving the performance of silicon solar cells. Adv. Mater..

[2] Xiang WC, Liu SZ, Tress W (2021). A review on the stability of inorganic metal halide perovskites: challenges and opportunities for stable solar cells. Energy Environ. Sci..

[3] Li JB (2021). Review on recent progress of lead-free halide perovskites in optoelectronic applications. Nano Energy.

[4] Sun R, Zhou DL, Song HW (2022). Rare earth doping in perovskite luminescent nanocrystals and photoelectric devices. Nano Sel..

[5] Wang YK (2021). All-inorganic quantum-dot LEDs based on a phase-stabilized α-CsPbI_3_ perovskite. Angew. Chem. Int. Ed..

[6] Liu Z (2021). Perovskite light-emitting diodes with EQE exceeding 28% through a synergetic dual-additive strategy for defect passivation and nanostructure regulation. Adv. Mater..

[7] Liu Y (2022). Wide-bandgap perovskite quantum dots in perovskite matrix for sky-blue light-emitting diodes. J. Am. Chem. Soc..

[8] Chen JW (2021). Perovskite white light emitting diodes: progress, challenges, and opportunities. ACS Nano.

[9] Chen H (2021). Efficient and bright warm-white electroluminescence from lead-free metal halides. Nat. Commun..

[10] Yao EP (2017). High-brightness blue and white LEDs based on inorganic perovskite nanocrystals and their composites. Adv. Mater..

[11] Mao J (2018). All-perovskite emission architecture for white light-emitting diodes. ACS Nano.

[12] Chen JW (2021). Efficient and bright white light-emitting diodes based on single-layer heterophase halide perovskites. Nat. Photonics.

[13] Chen ZM (2021). Utilization of trapped optical modes for white perovskite light-emitting diodes with efficiency over 12%. Joule.

[14] Pan GC (2017). Doping lanthanide into perovskite nanocrystals: highly improved and expanded optical properties. Nano Lett..

[15] Zhou DL (2019). Impact of host composition, codoping, or tridoping on quantum-cutting emission of ytterbium in halide perovskite quantum dots and solar cell applications. Nano Lett..

[16] Sun R (2020). Samarium-doped metal halide perovskite nanocrystals for single-component electroluminescent white light-emitting diodes. ACS Energy Lett..

[17] Puntus LN (2002). Charge transfer bands in the Eu^3+^ luminescence excitation spectra of isomeric europium pyridine-dicarboxylates. Phys. Solid State.

[18] Wang R (2019). Constructive molecular configurations for surface-defect passivation of perovskite photovoltaics. Science.

[19] Begum R (2017). Engineering interfacial charge transfer in CsPbBr_3_ perovskite nanocrystals by heterovalent doping. J. Am. Chem. Soc..

[20] Van Der Stam W (2017). Highly emissive divalent-ion-doped colloidal CsPb_1–*x*_M_*x*_Br_3_ perovskite nanocrystals through cation exchange. J. Am. Chem. Soc..

[21] Yang JN (2020). Potassium bromide surface passivation on CsPbI_3-*x*_Br_*x*_ nanocrystals for efficient and stable pure red perovskite light-emitting diodes. J. Am. Chem. Soc..

[22] Nam JK (2017). Potassium incorporation for enhanced performance and stability of fully inorganic cesium lead halide perovskite solar cells. Nano Lett..

[23] Yu ZL (2020). Theoretical study on the effect of the optical properties and electronic structure for the Bi-doped CsPbBr_3_. J. Phys. Condens. Matter.

[24] Zhang JY (2019). Disappeared deep charge-states transition levels in the p-type intrinsic CsSnCl_3_ perovskite. Appl. Phys. Lett..

[25] Kang J, Wang LW (2017). High defect tolerance in lead halide perovskite CsPbBr_3_. J. Phys. Chem. Lett..

[26] Kayanuma Y, Momiji H (1990). Incomplete confinement of electrons and holes in microcrystals. Phys. Rev. B.

[27] Brus LE (1983). A simple model for the ionization potential, electron affinity, and aqueous redox potentials of small semiconductor crystallites. J. Chem. Phys..

[28] Kong DY (2015). Spectroscopic studies on interaction of BSA and Eu(III) complexes with H_5_ph-dtpa and H_5_dtpa ligands. Spectrochim. Acta A Mol. Biomol. Spectrosc..

[29] Tang J (2011). Colloidal-quantum-dot photovoltaics using atomic-ligand passivation. Nat. Mater..

[30] Zhou DL (2017). Semiconductor plasmon induced up-conversion enhancement in mCu_2–*x*_S@SiO_2_@Y_2_O_3_: Yb^3+^/Er^3+^ core–shell nanocomposites. ACS Appl. Mater. Interfaces.

[31] Prodi L (1991). Luminescence properties of cryptate europium (III) complexes incorporating heterocyclic N-oxide groups. Chem. Phys. Lett..

[32] Karki KJ (2016). Different emissive states in the bulk and at the surface of methylammonium lead bromide perovskite revealed by two-photon micro-spectroscopy and lifetime measurements. APL Photonics.

[33] Han CF (2018). Unraveling surface and bulk trap states in lead halide perovskite solar cells using impedance spectroscopy. J. Phys. D Appl. Phys..

[34] Yang Y (2015). Low surface recombination velocity in solution-grown CH_3_NH_3_PbBr_3_ perovskite single crystal. Nat. Commun..

[35] Huang SQ (2018). Postsynthesis potassium-modification method to improve stability of CsPbBr_3_ perovskite nanocrystals. Adv. Optical Mater..

[36] Palazon F (2016). X-ray lithography on perovskite nanocrystals films: from patterning with anion-exchange reactions to enhanced stability in air and water. ACS Nano.

[37] De Quilettes DW (2015). Impact of microstructure on local carrier lifetime in perovskite solar cells. Science.

[38] Wu SF (2020). Modulation of defects and interfaces through alkylammonium interlayer for efficient inverted perovskite solar cells. Joule.

[39] Huang H (2017). Lead halide perovskite nanocrystals in the research spotlight: stability and defect tolerance. ACS Energy Lett..

[40] Hu QK (2021). Dual defect-passivation using phthalocyanine for enhanced efficiency and stability of perovskite solar cells. Small.

[41] Chung Y, Kim KS, Jung JW (2022). On the role of carboxylated polythiophene in defect passivation of CsPbI_2_Br surface for efficient and stable all-inorganic perovskite solar cells. Int. J. Energy Res..

[42] Lin H (2021). Stable and efficient blue-emitting CsPbBr_3_ nanoplatelets with potassium bromide surface passivation. Small.

[43] Kong LM (2021). Smoothing the energy transfer pathway in quasi-2D perovskite films using methanesulfonate leads to highly efficient light-emitting devices. Nat. Commun..

[44] Ma DX (2021). Distribution control enables efficient reduced-dimensional perovskite LEDs. Nature.

[45] Yang S (2019). Tailoring passivation molecular structures for extremely small open-circuit voltage loss in perovskite solar cells. J. Am. Chem. Soc..

[46] Ma CQ, Park NG (2020). Paradoxical approach with a hydrophilic passivation layer for moisture-stable, 23% efficient perovskite solar cells. ACS Energy Lett..

[47] Dong QF (2015). Electron-hole diffusion lengths >175 μm in solution-grown CH3NH3PbI3 single crystals. Science.

[48] Liu ZH (2017). Chemical reduction of intrinsic defects in thicker heterojunction planar perovskite solar cells. Adv. Mater..

[49] Lu M (2020). Bright CsPbI_3_ perovskite quantum dot light-emitting diodes with top-emitting structure and a low efficiency roll-off realized by applying zirconium acetylacetonate surface modification. Nano Lett..

[50] Park MH (2019). Boosting efficiency in polycrystalline metal halide perovskite light-emitting diodes. ACS Energy Lett..

[51] Sanehira EM (2017). Enhanced mobility CsPbI_3_ quantum dot arrays for record-efficiency, high-voltage photovoltaic cells. Sci. Adv..

[52] Vickers ET (2020). Enhancing charge carrier delocalization in perovskite quantum dot solids with energetically aligned conjugated capping ligands. ACS Energy Lett..

[53] Soreni-Harari M (2008). Tuning energetic levels in nanocrystal quantum dots through surface manipulations. Nano Lett..

[54] Zhang XY (2018). PbS capped CsPbI_3_ nanocrystals for efficient and stable light-emitting devices using *p–i–n* structures. ACS Cent. Sci..

[55] Wood V (2010). Air-stable operation of transparent, colloidal quantum dot based LEDs with a unipolar device architecture. Nano Lett..

[56] Sun QJ (2007). Bright, multicoloured light-emitting diodes based on quantum dots. Nat. Photonics.

[57] Anikeeva PO (2009). Quantum dot light-emitting devices with electroluminescence tunable over the entire visible spectrum. Nano Lett..

[58] Senawiratne J (2008). Junction temperature analysis in green light emitting diode dies on sapphire and GaN substrates. Phys. Status Solidi C..

[59] Lu YF, Cao XA (2014). Blue and green electroluminescence from CdSe nanocrystal quantum-dot-quantum-wells. Appl. Phys. Lett..

[60] Hasan T (2018). Photo-and electroluminescence from nitrogen-doped and nitrogen–sulfur codoped graphene quantum dots. Adv. Funct. Mater..

[61] Song YL (2017). White electroluminescence from a prototypical light-emitting diode based on CdS/Si heterojunctions. Mater. Lett..

