# Supplementary material for: Efficient single-component white light emitting diodes enabled by lanthanide ions doped lead halide perovskites via controlling Förster energy transfer and specific defect clearance
Source: Light Sci Appl. 2022 Dec 6;11:340. doi: 10.1038/s41377-022-01027-9 (PMC9722690; doi:10.1038/s41377-022-01027-9)
Supplement: Supplementary file 1 — supplementary information [file 41377_2022_1027_MOESM1_ESM.docx]

**Supplementary Information**

**Efficient Single-Component White Light Emitting Diodes** **Enabled by Lanthanide Ions Doped Lead Halide Perovskites via** **Controlling Förster Energy Transfer and Specific Defect Clearance**

*Rui Sun, Donglei Zhou*, Yujiao Ding, Yue Wang, Yuqi Wang, Xinmeng Zhuang, Shuainan Liu, Nan Ding, Tianyuan Wang, Wen Xu, Hongwei Song**

**Supplementary Note 1**

DFT calculations

All our theoretical calculations were carried out by employing the first-principles simulations based on density functional theory (DFT) within the Vienna Ab-initio Simulation Package (VASP) 1-2. The projector augmented wave (PAW) method was used to describe the electron–ion interaction and the exchange-correlation between electrons was described by the generalized gradient approximation (GGA) in the Perdew–Burke–Ernzerhof (PBE) form 3-4. A cutoff energy of 500 eV was used for the plane-wave basis set in all calculations. A supercell of (3×3×3) unit cell of bulk CsPbCl_3_, with one Pb atom replaced by Eu atom, was employed to model the doped structure. The Brillouin-zone integrations were performed using a (2×2×2) k-mesh of the Monkhorst-Pack sampling scheme5. All the atoms in the model were relaxed until the force on each atom is below 0.01 eV Å^-1^.

For the doping of K atom, three different sites were considered. They are interstitial site, Pb-substantial site, and Cs-substantial site, respectively. We have calculated the formation energy of the Eu and K co-doped CsPbCl_3_, where the K atom is near the Eu atom, as follow:

Ef = E(Eu-K- CsPbCl_3_) – E(CsPbCl_3_) – E(Eu) – E(K)

where E(Eu-K- CsPbCl_3_), E(CsPbCl_3_), E(Eu) and E(K) are the total energy of the Eu and K co-doped CsPbCl_3_, pure CsPbCl_3_, elemental Eu and K, respectively. A negative value of E_f_ means the structure is energetical stable.

**Supplementary Note 2**

Calculation of radiative & non-radiative decay rate

Radiative lifetime: 𝜏_𝑟_ = <𝑡>/PLQY;

Nonradiative lifetime: 𝜏_𝑛𝑟_ = <𝑡>/(1-PLQY);

Radiative decay rate: 𝑘_𝑟_ = 1/ 𝜏_𝑟_;

Nonradiative decay rate: 𝑘_𝑛𝑟_ = 1/ 𝜏_𝑛𝑟_;

**Supplementary Note 3**

Spectral overlap simulation

The PL emission curves of the perovskite host was set as a Gaussian curve with FWHM of 15 nm, peak of 1 and emission center of x_c_ (x_c_ = 394~414 nm). The absorption band ^7^F_0_-^5^L_6_ of Eu^3+^ ions was set as a Gaussian curve with FWHM of 5 nm, peak of 1 and emission center of 394 nm.

These tow curves are represented as follows:

y_c_ = exp((-4ln2(x-x_c_)^2^)/15^2^)

y = exp((-4ln2(x-394)^2^)/5^2^)

**Supplementary Figures and Tables:**


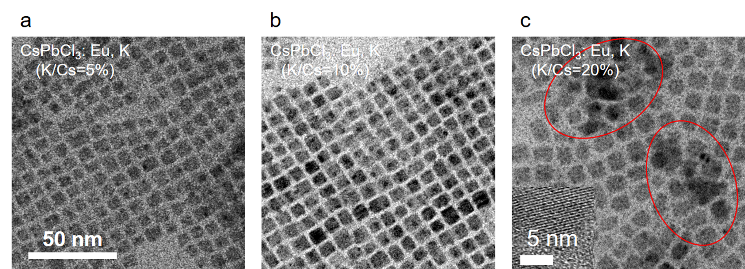


Figure S1. TEM images of CsPbCl_3_: Eu^3+^ with K^+^ (K/Cs=5, 10, 20%) ion codoped (inset of c: HR-TEM for CsPbCl_3_: Eu^3+^ with 10% K^+^).


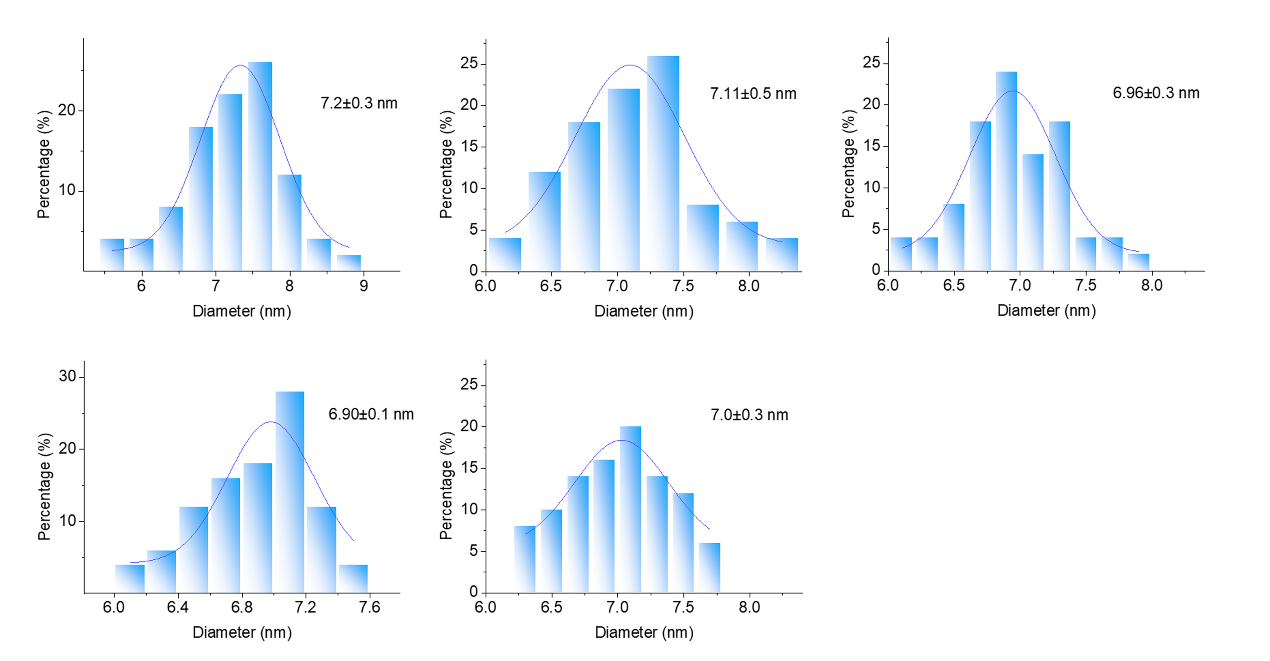


Figure S2. The size distribution of CsPbCl_3_: Eu^3+^ codoped with various concentration of K^+^ (K/Cs=0-20%) ions.


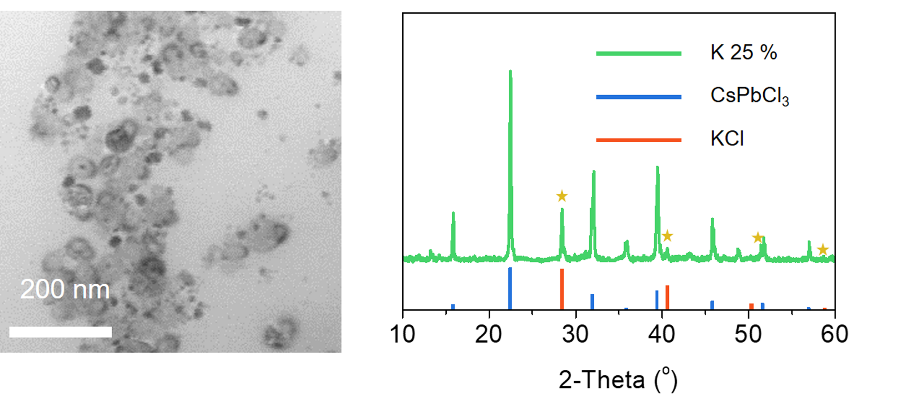


Figure S3. TEM image and XRD pattern of CsPbCl_3_: Eu^3+^ with 25% K^+^ ion doping concentration, respectively.


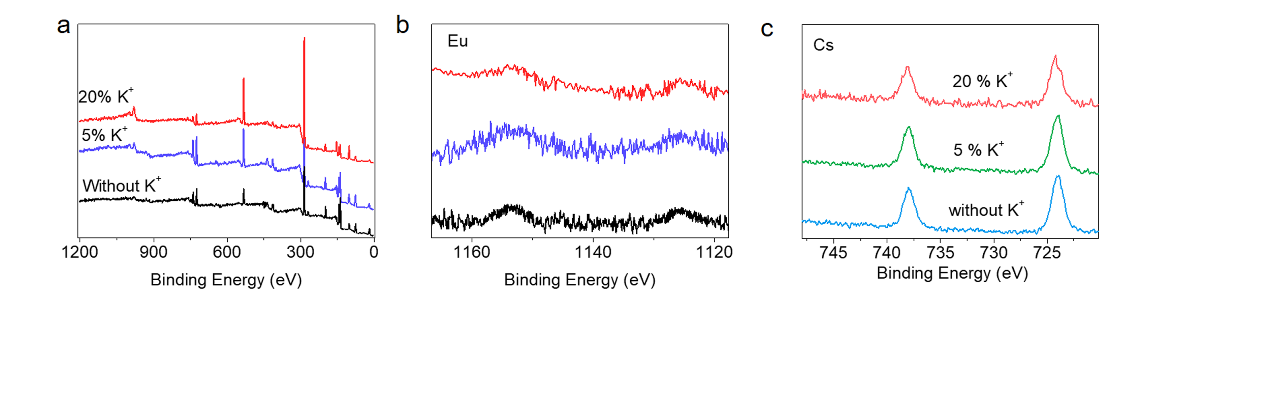


Figure S4. (a) Survey XPS spectra for the CsPbCl_3_: Eu^3+^ PeNCs with K^+^ ion doping concentration of 0, 5 and 20%. (b, c) High-resolution XPS analysis corresponding to Eu 3d_3/2_ and 3d_5/2_, Cs 3d in CsPbCl_3_: Eu^3+^ PeNCs with K^+^ ion doping concentration of 0, 5 and 20%.


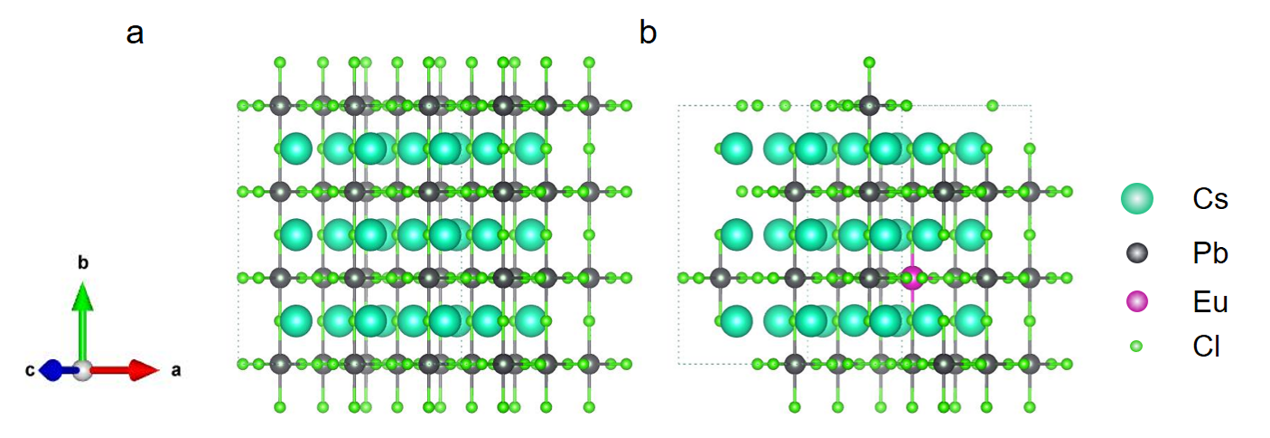


Figure S5. Structure models of (a) CsPbCl_3_ (b) CsPb_0.93_Eu_0.07_Cl_3_ PeNCs.


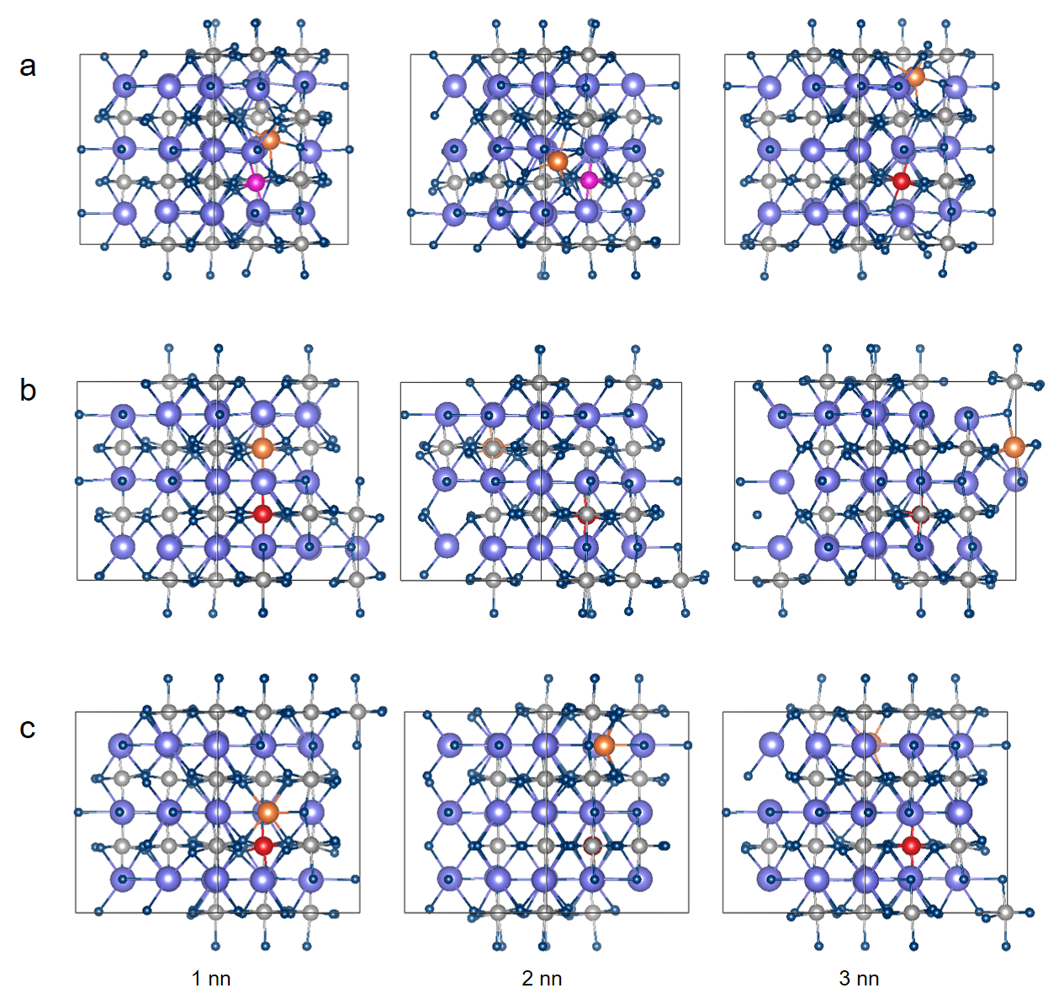


Figure S6. Structure models of CsPbCl_3_ codoped with Eu^3+^ and K^+^, in which the K^+^ occupied interstitial site (a), Pb-substantial site (b), and Cs-substantial site (c), respectively.


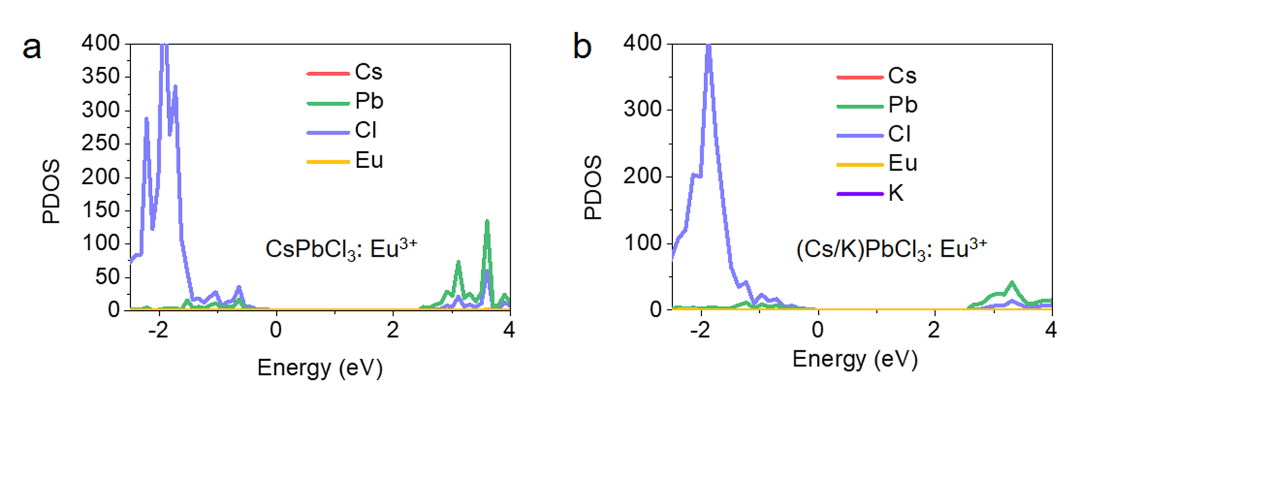


Figure S7. The DOS for CsPbCl_3_: Eu^3+^ and (Cs/K)PbCl_3_: Eu^3+^, respectively.


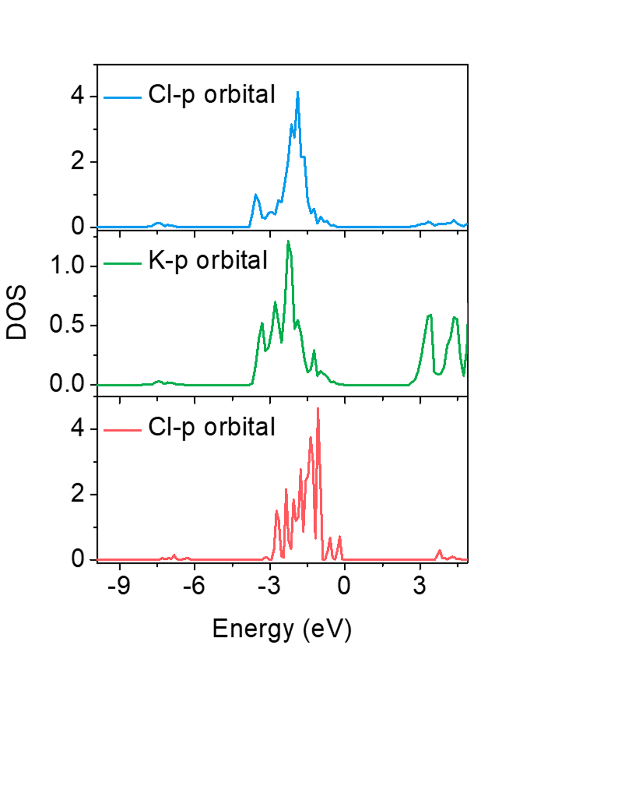


Figure S8. The DOS of p orbital in Cl atom for the CsPbCl_3_: Eu^3+^ (red line) and (Cs/K)PbCl_3_: Eu^3+^ (blue line), and p orbital in K atom for the (Cs/K)PbCl_3_: Eu^3+^ (green line) , respectively. (The chosen Cl atom is around K atom)


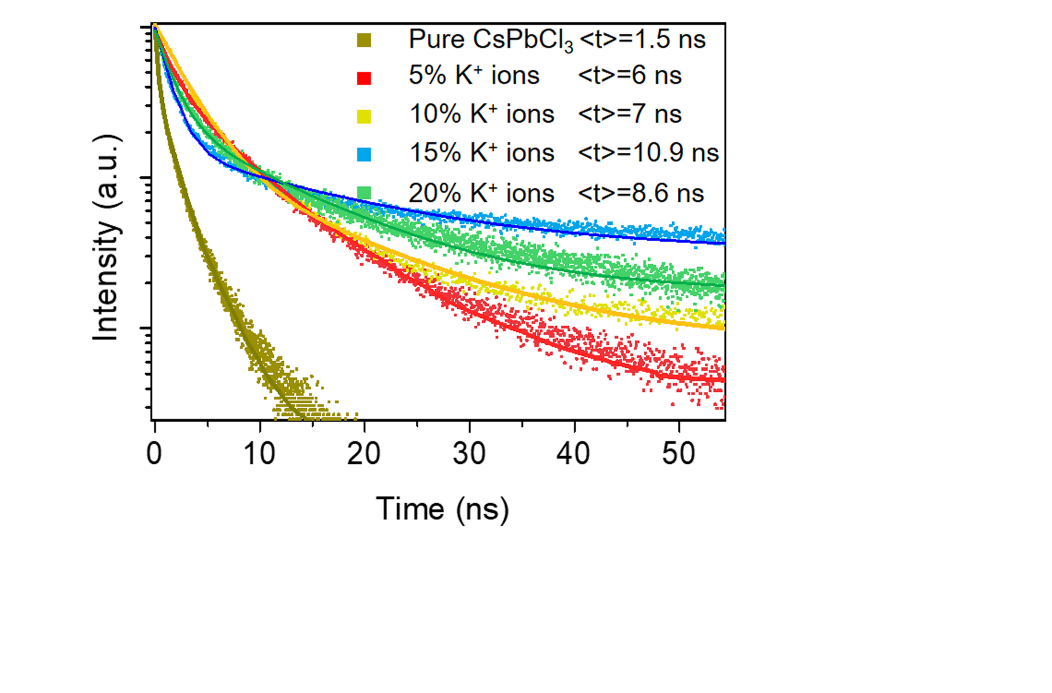


Figure S9. Emission decay curves for the CsPbCl_3_ PeNCs doped with various K^+^ ion concentration acquired by monitoring the respective excitonic emission.


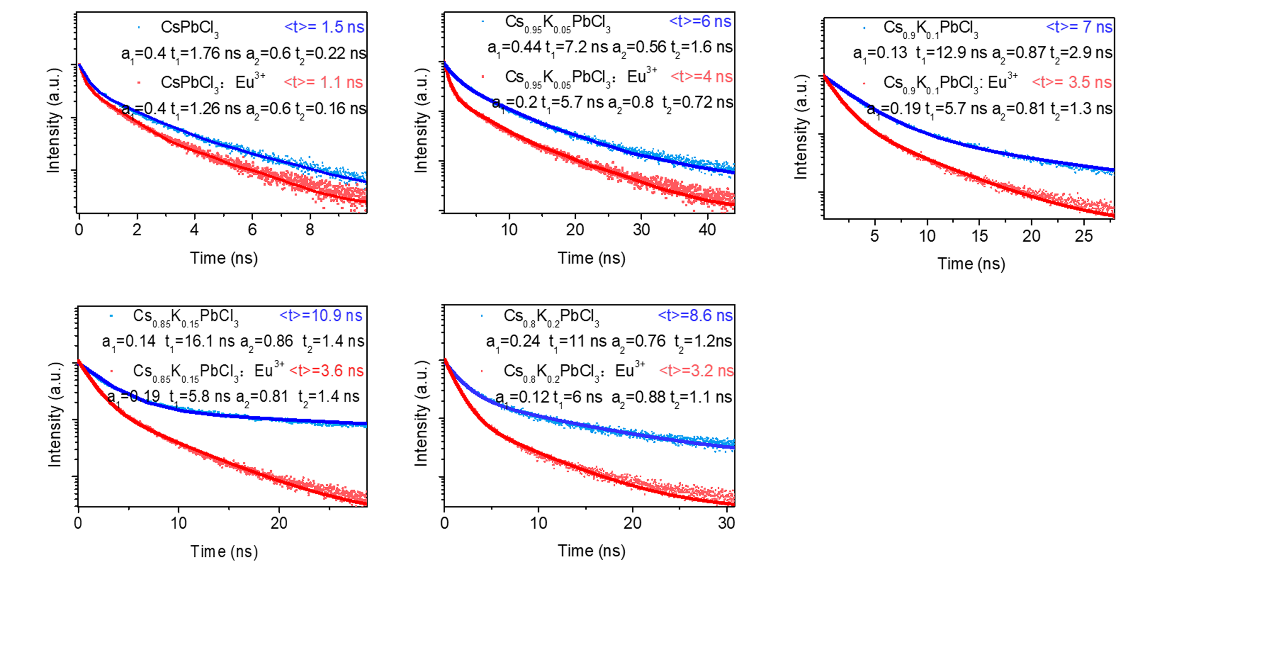


Figure S10. Excitonic emission decay curves of CsPbCl_3_, CsPbCl_3_: Eu^3+^ PeNCs with various K^+^ ion doping concentrations.


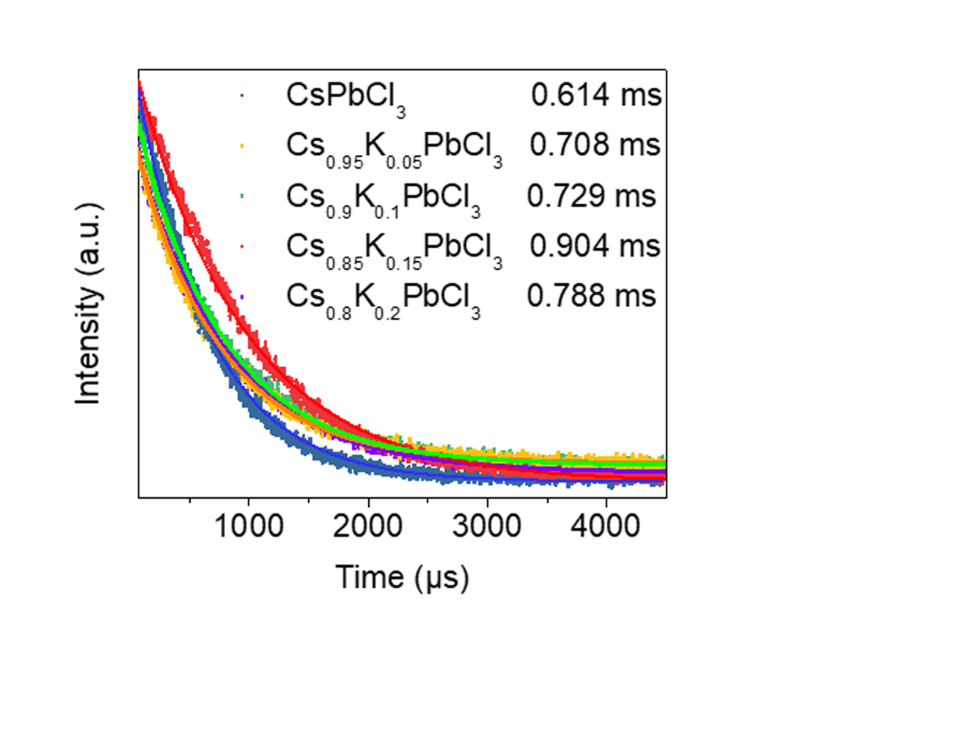


Figure S11. Emission dynamics for CsPbCl_3_: Eu^3+^ PeNCs with various K^+^ ion doping concentrations acquired by monitoring at 622 nm.


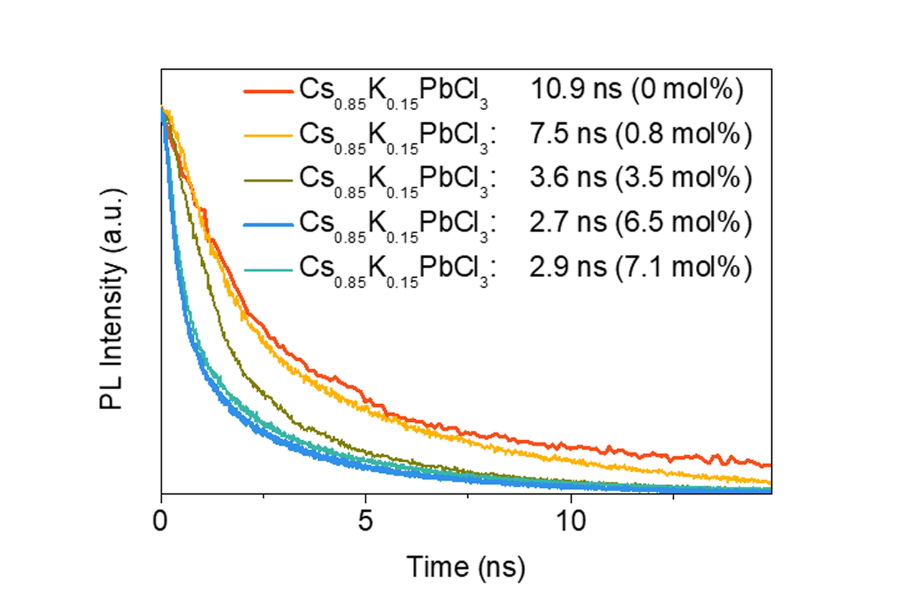


Figure S12. Excitonic emission decay curves of Cs_0.85_K_0.15_PbCl_3_ PeNCs with various Eu^3+^ ion doping concentrations.


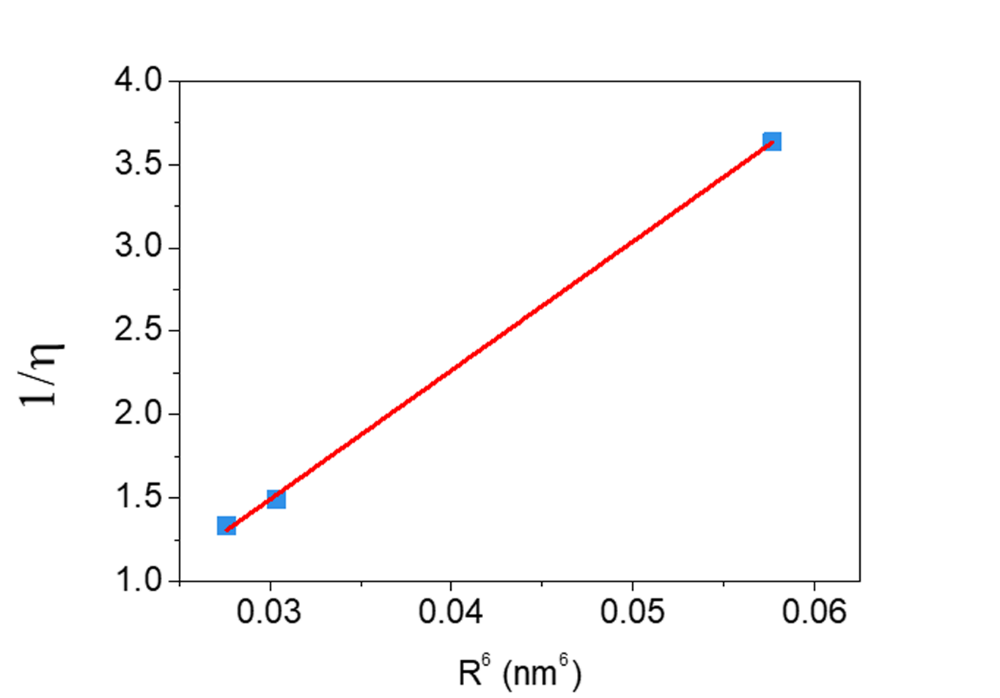


Figure S13. Dependence of 1/η and R^6^. R: distance between the donor and acceptor.


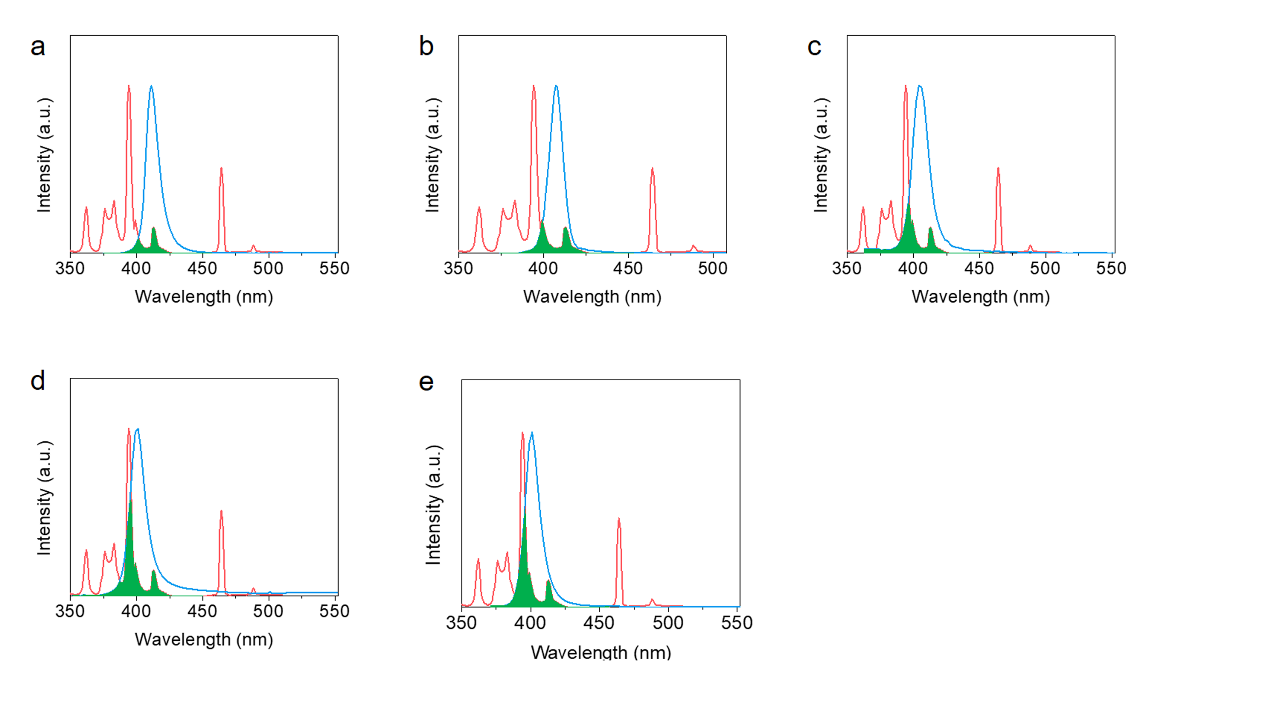


Figure S14. Absorption spectrum of Eu^3+^ ions (red line) and PL spectra of Cs_1-x_K_x_PbCl_3_ (blue line).


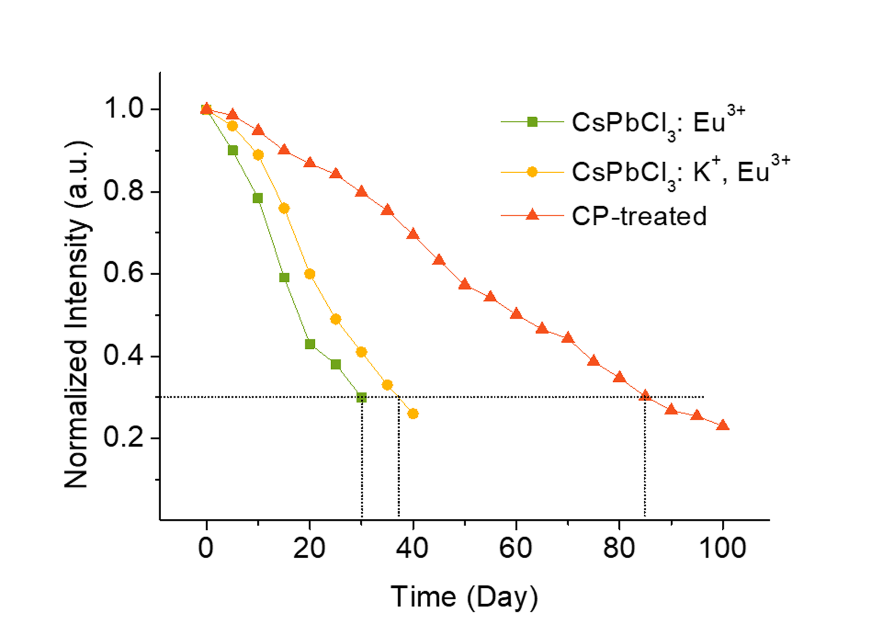


Figure S15. The stability of PL fromCsPbCl_3_: Eu^3+^ and CsPbCl_3_: K^+^, Eu^3+^ PeNC film, CP-treated CsPbCl_3_: K^+^, Eu^3+^ PeNC film, excited by 365 nm under the atmosphere condition.


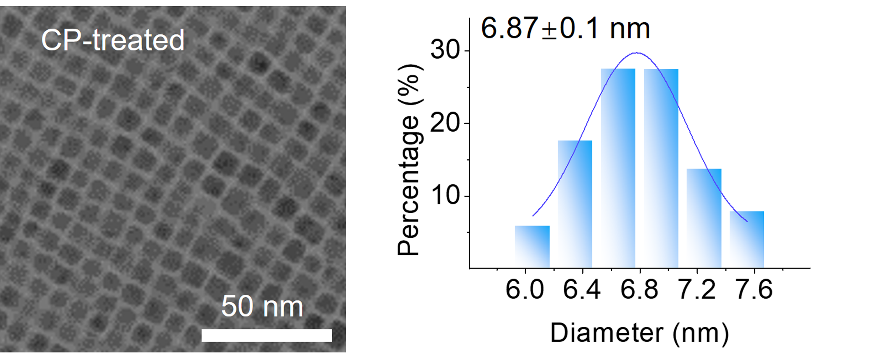


Figure S16. TEM images and size distribution for CP-treated PeNCs.


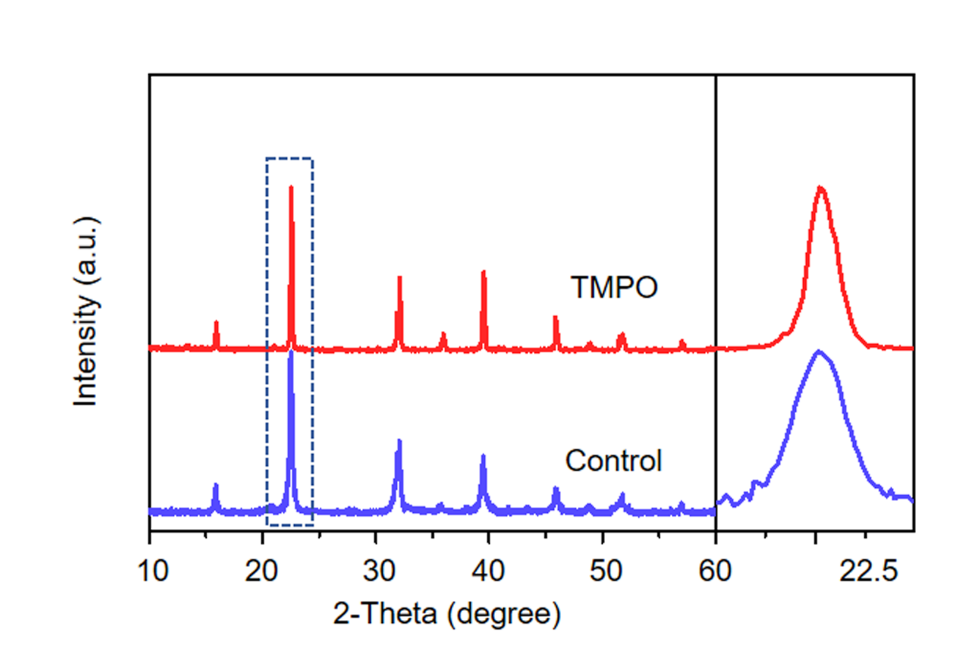


Figure S17. XRD patterns of the control PeNC film and treated film, respectively.


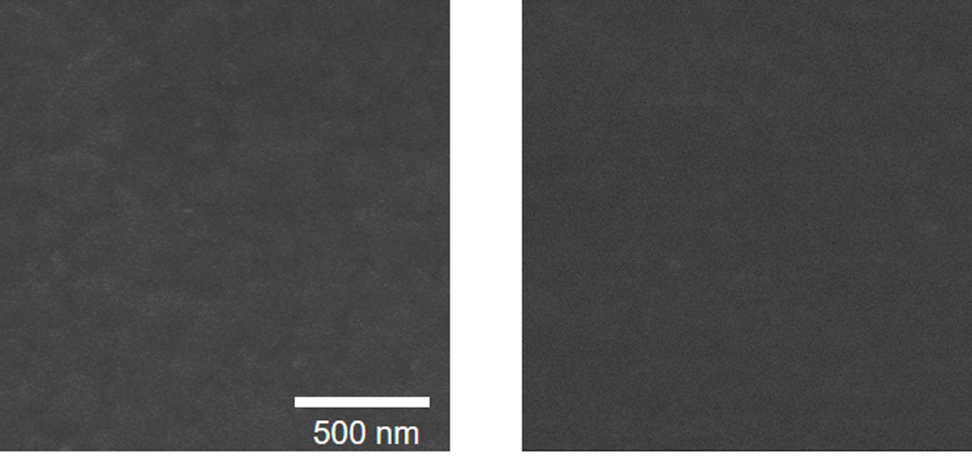


Figure S18. SEM images of pristine (left) and modified (right) CsPbCl_3_: Eu^3+^, K^+^ PeNCs, respectively.


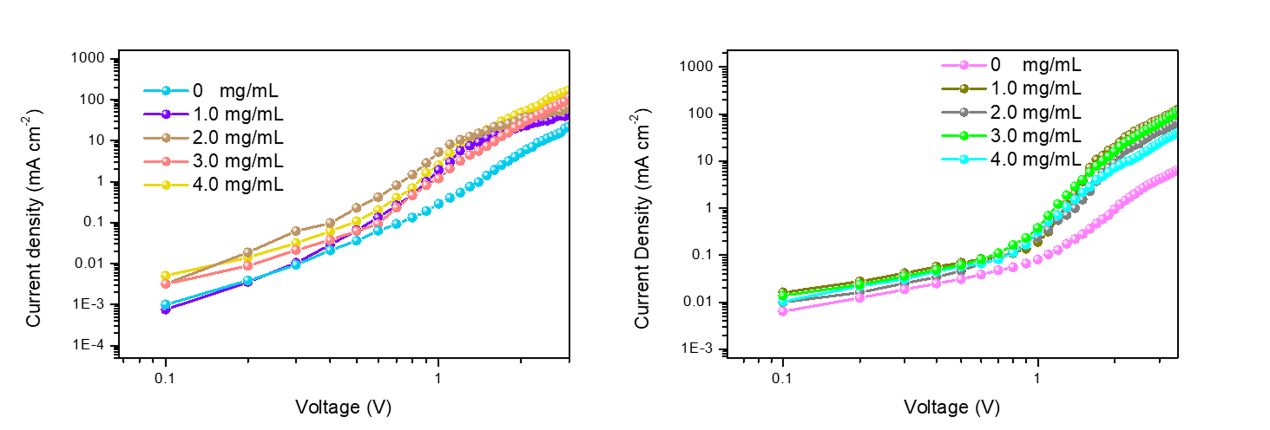


Figure S19. The electron-only devices and (left) hole-only devices (right) of PeNCs treated with various concentrations of CP.


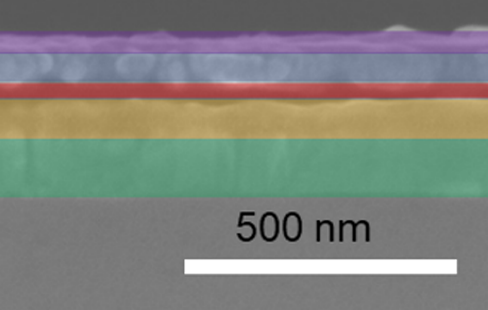


Figure S20. SEM image of the cross section for the device.


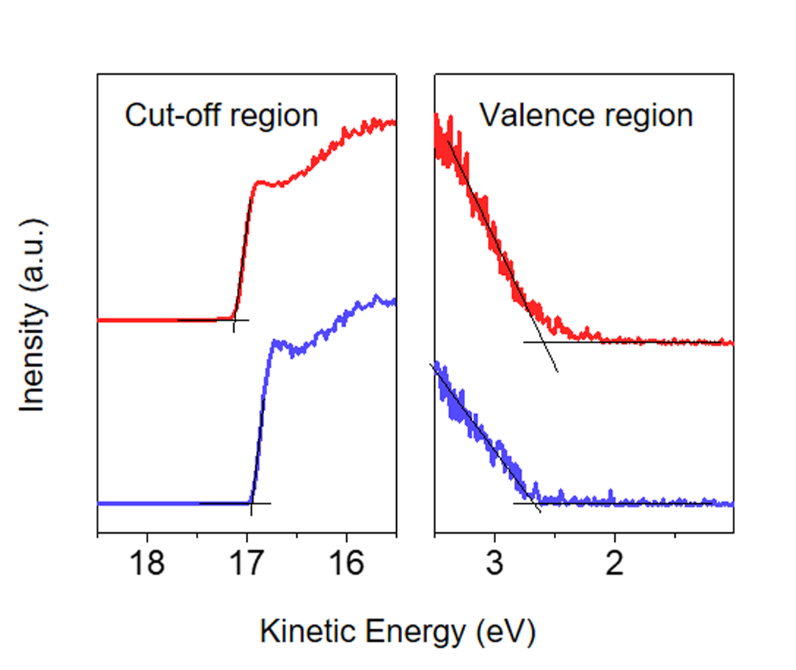


Figure S21. UPS spectra of pristine (blue line) and modified (red line) CsPbCl_3_: Eu^3+^, K^+^ PeNCs, respectively.


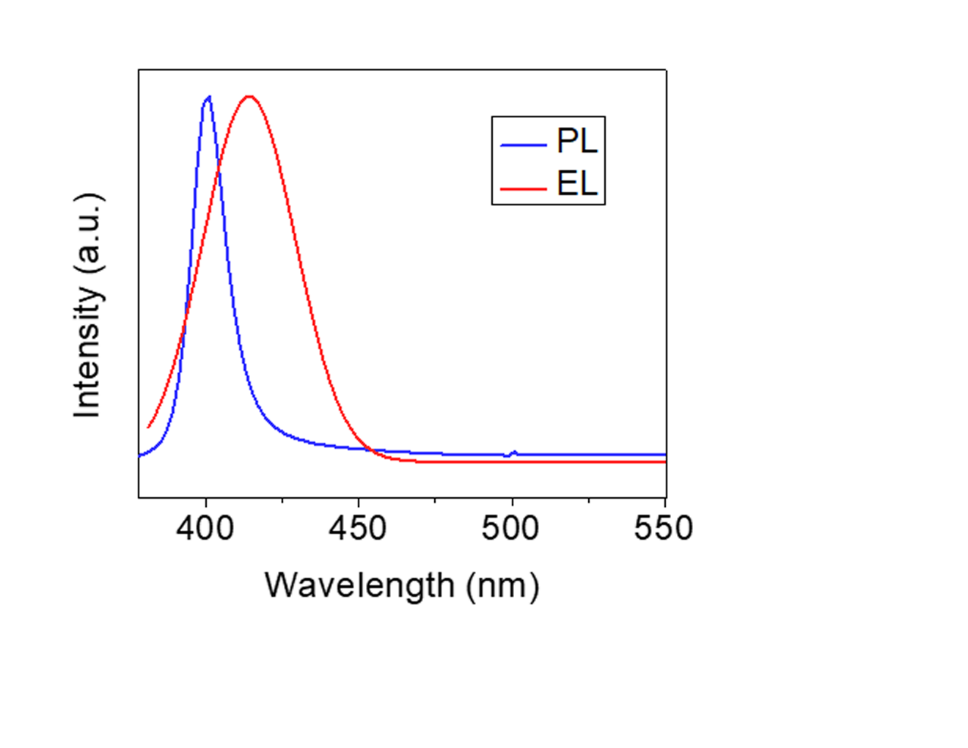


Figure S22. Excitonic spectra comparison of PL of PeNC solution versus EL of perovskite LED.


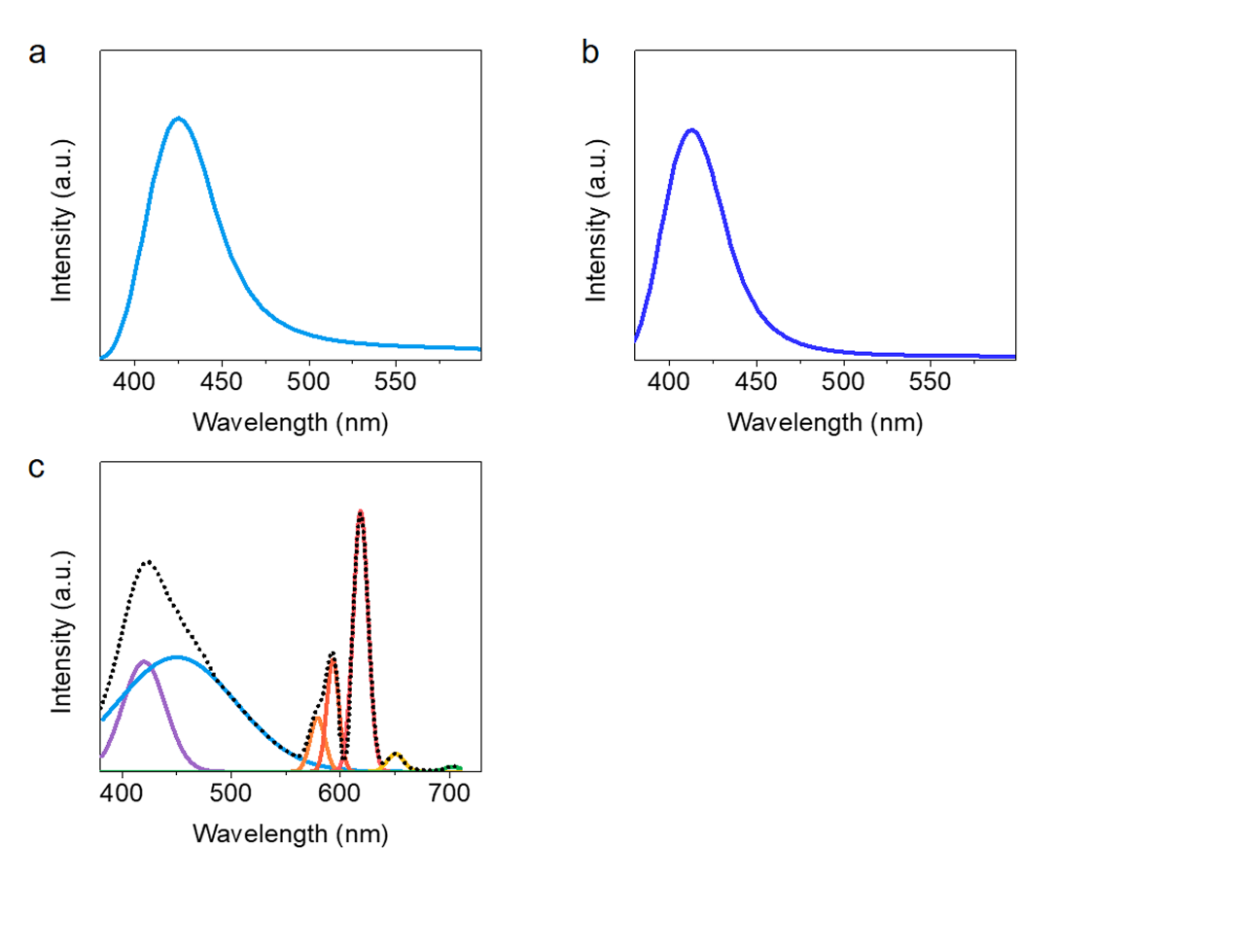


Figure S23. EL spectra of PeLEDs based on CsPbCl_3_, Cs_0.85_K_0.15_PbCl_3_ and CsPbCl_3_: Eu^3+^ PeNCs, fitted by the Gaussian functions.


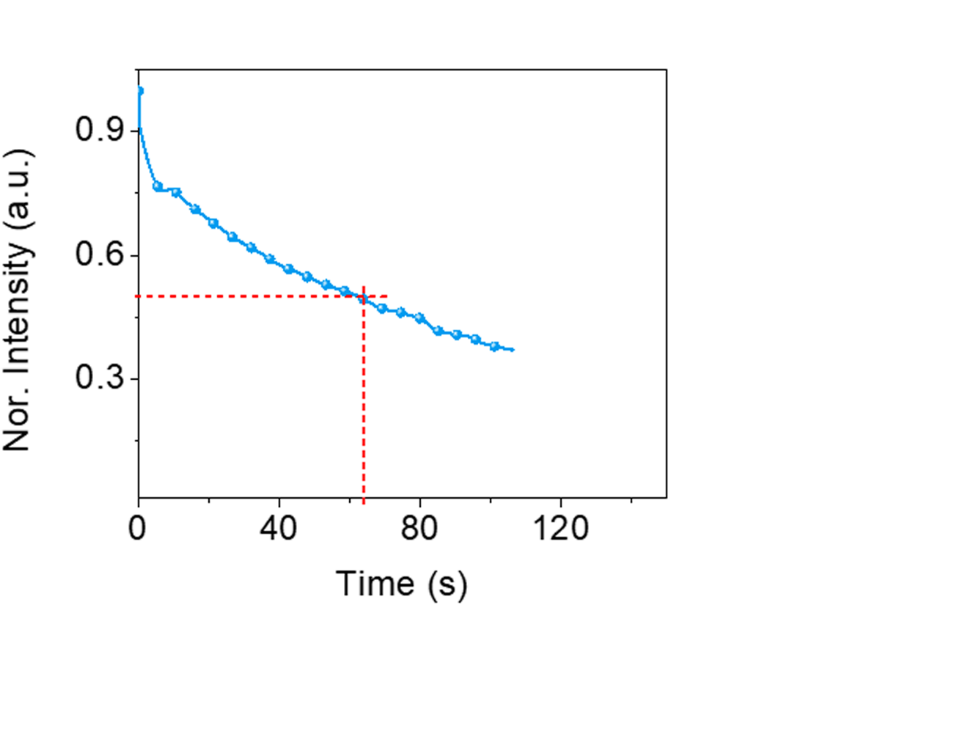


Figure S24. Operational stability lifetime of LEDs based on 3.5 mol% Eu^3+^ ion doping concentration in CsPbCl_3_ PeNCs without K^+^ ions and CP modified.


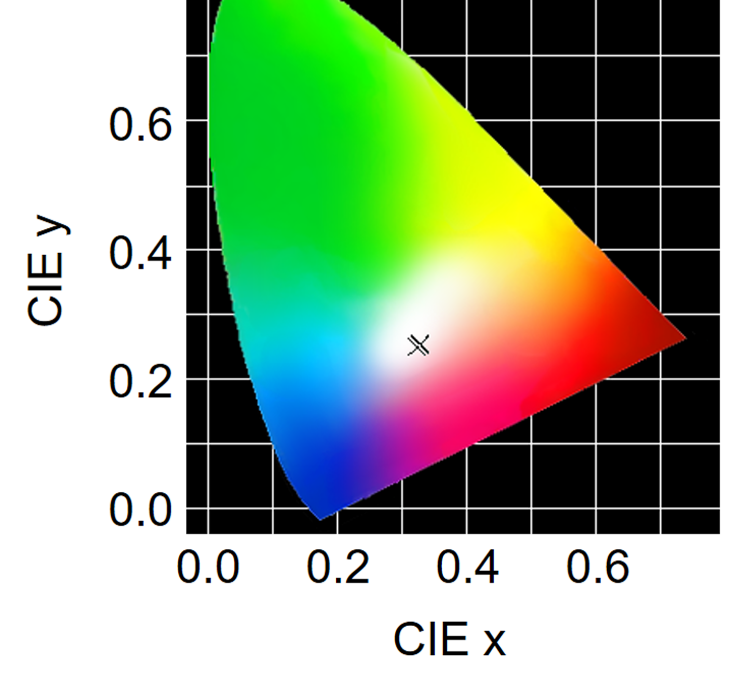


Figure S25. CIE coordinates against voltage change of white LED.


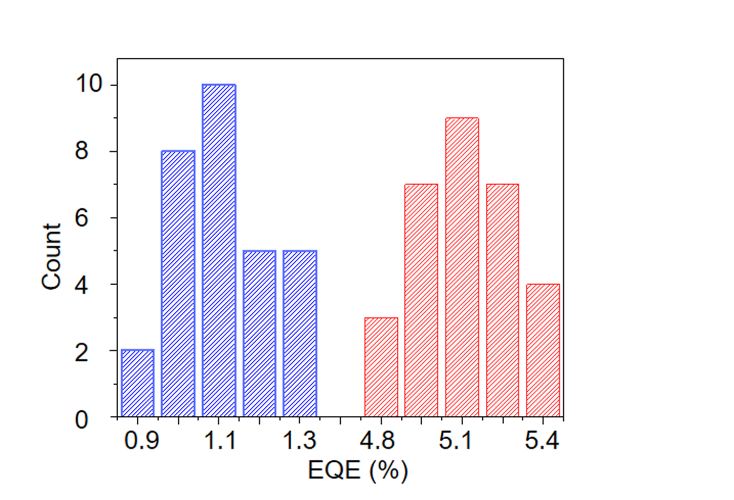


Figure S26. EQE histogram of 30 devices based on control, CP-treated PeNCs, 3.5 mol% Eu^3+^ ion doping concentration.


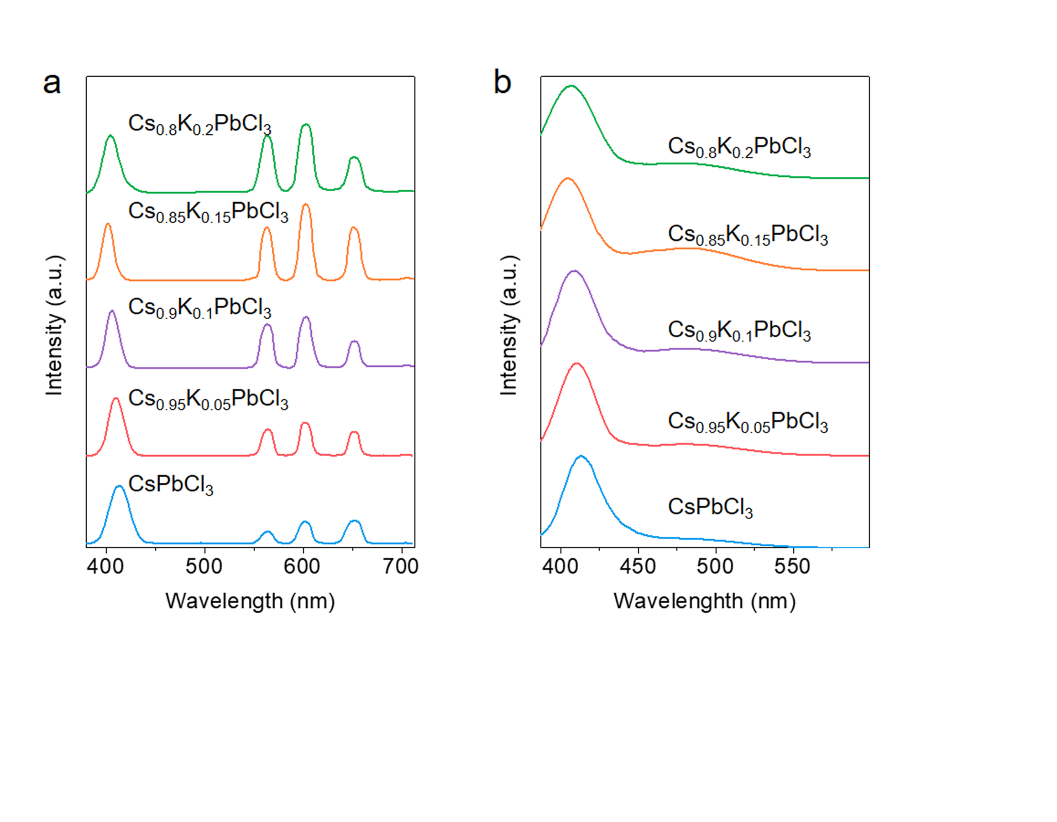


Figure S27. The PL spectra of the Cs_1-x_K_x_PbCl_3_ PeNCs (x=0-0.2) doped with Sm^3+^ (5.1 mol%), Er^3+^ ions (1.8 mol%).


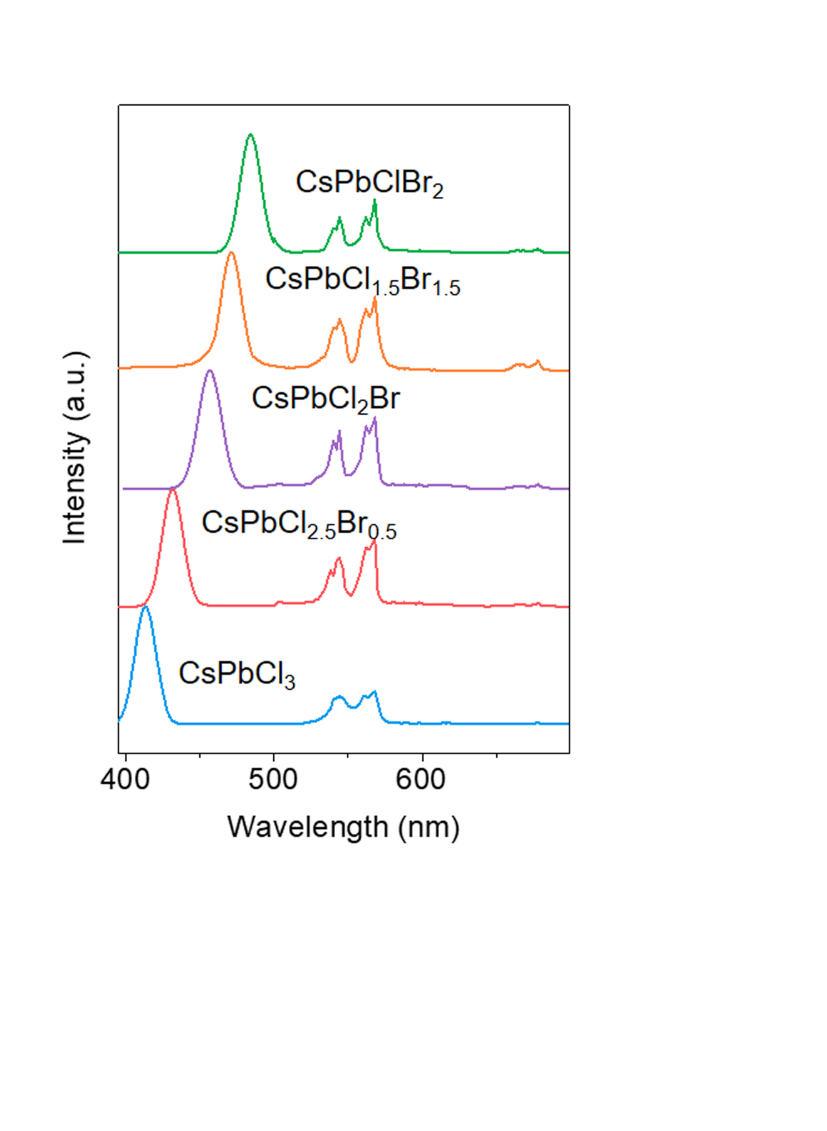


Figure S28. The PL spectra of the CsPb(Cl/Br)_3_ PeNCs doped with Er^3+^ ions (3.1 mol%).


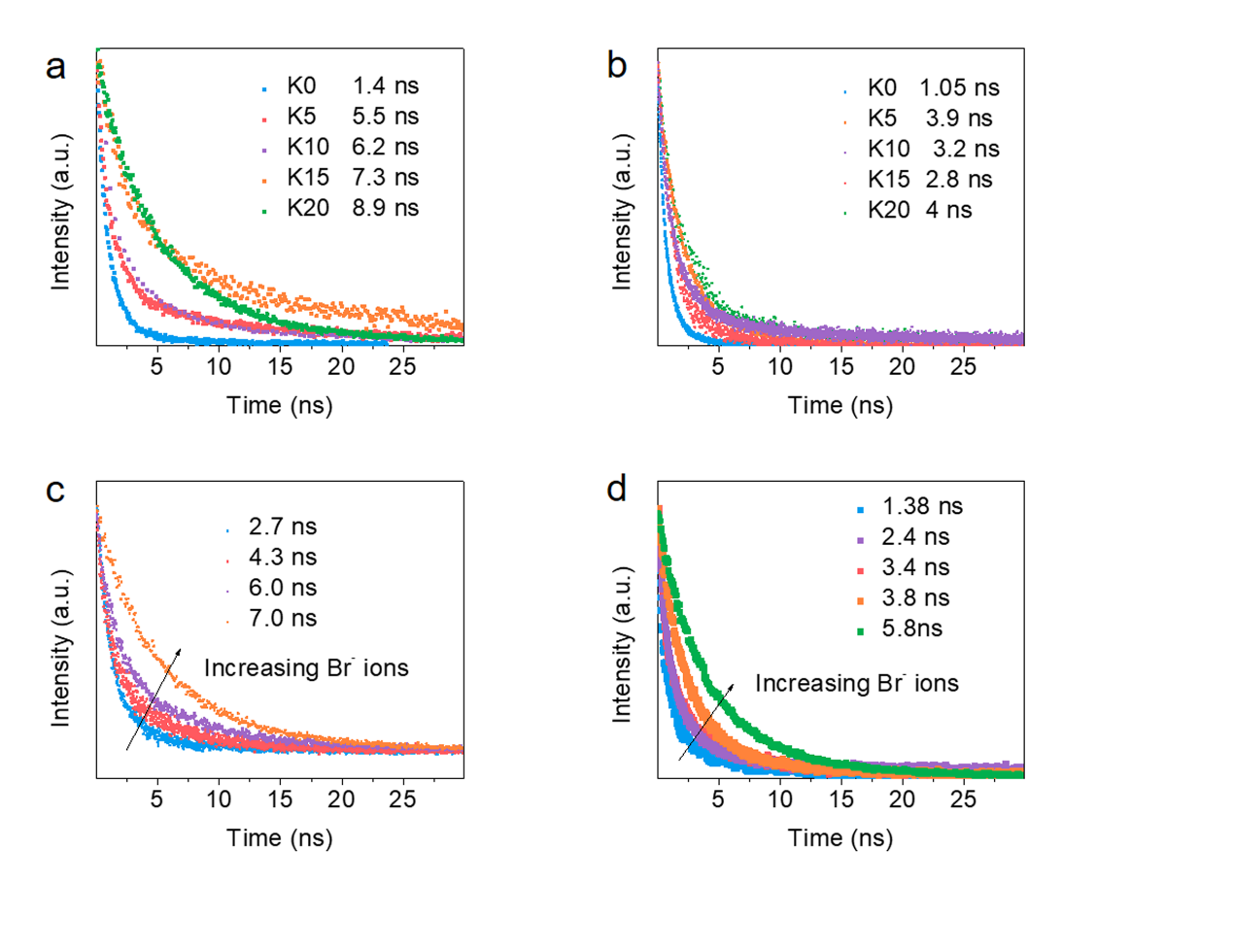


Figure S29. (a-b) The emission decay curves for the Cs_1-x_K_x_PbCl_3_ PeNCs (x=0-0.2) doped with Sm^3+^ (5.1 mol%), Ce^3+^ (1.8 mol%) ions, respectively. (c) The emission decay curves for the CsPb(Cl/Br)_3_ PeNCs doped without and with Er^3+^ ions (3.1 mol%).


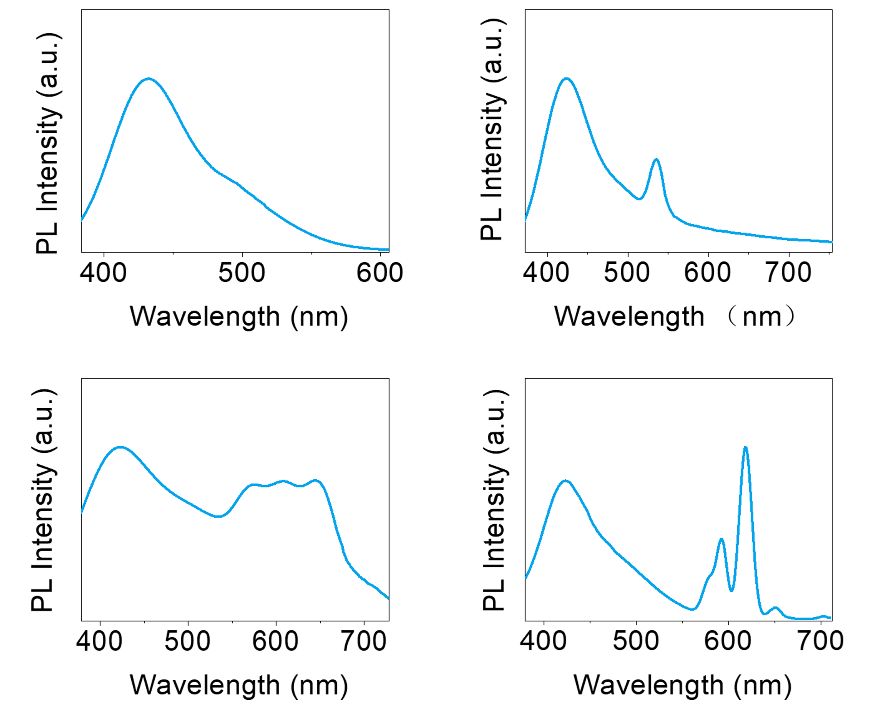


Figure S30. The EL spectra for the LEDs based on various Ln^3+^ ions doped CsPbCl_3_ PeNCs before energy band regulation and defect passivation.

Table S1. Actual doping concentrations of Eu^3+^ and K^+^ in K^+^ doped CsPbCl_3_: Eu^3+^ PeNCs measured by ICP-MS.

| Eu/Pb (%) | 5 | | 10 | | 15 | |
| --- | --- | --- | --- | --- | --- | --- |
| K/Cs (%) | K (mol%) | Eu (mol%) | K (mol%) | Eu (mol%) | K (mol%) | Eu (mol%) |
| 0 | 0 | 0.7 | 0 | 3.5 | 0 | 6.8 |
| 5 | 3.2 | 0.7 | 3.4 | 3.7 | 3.1 | 6.7 |
| 10 | 4.7 | 0.8 | 5.0 | 3.6 | 4.8 | 7.0 |
| 15 | 7.0 | 0.8 | 6.9 | 3.5 | 6.6 | 6.8 |
| 20 | 12.9 | 0.7 | 12.7 | 3.7 | 12.3 | 6.5 |

Table S2. The lattice constant of Cs_1-x_K_x_PbCl_3_ PeNCs doped with Eu^3+^ ions (3.5 mol%).

| Samples | CsPbCl_3_ | Cs_0.95_K_0.05_PbCl_3_ | Cs_0.9_K_0.1_PbCl_3_ | Cs_0.85_K_0.15_PbCl_3_ | Cs_0.8_K_0.2_PbCl_3_ |
| --- | --- | --- | --- | --- | --- |
| Lattice constant  (110) (Å) | 3.97 | 3.95 | 3.94 | 3.92 | 3.94 |
| Average size (nm) | 7.20 | 7.11 | 6.96 | 6.90 | 7.04 |
| Bandgap  (eV) | 2.95 | 2.97 | 3.01 | 3.07 | 3.02 |
| ΔE  (meV) | _ | 1.5 | 4.33 | 6 | 2.93 |

Table S3. Overall PLQY, PLQY associated with the intrinsic transitions of Eu^3+^ ions, and PLQY associated with the excitonic transition as a function of the samples with different K^+^ ion concentrations.

| K^+^ concentration | 0% | 5% | 10% | 15% | 20% |
| --- | --- | --- | --- | --- | --- |
| PLQY of Exciton | 18% | 26% | 16% | 22% | 18% |
| PLQY of Eu^3+^ | 2% | 14% | 40% | 55% | 47% |
| Total PLQY | 20% | 40% | 56% | 77% | 65% |

Table S4. Emission decay curves of excitonic transitions for PeNCs doped with various K^+^ ion doping concentrations.

| K^+^ ion doping concentration (%) | 20 | 15 | 10 | 5 | 0 |
| --- | --- | --- | --- | --- | --- |
| A_1_ | 0.24 | 13.46 | 0.13 | 0.44 | 0.4 |
| τ_1_ (ns) | 11.02 | 16.11 | 12.9 | 7.23 | 1.76 |
| A_2_ | 0.76 | 86.54 | 0.87 | 0.56 | 0.6 |
| τ_2_ (ns) | 1.1618 | 1.35 | 2.92 | 1.6 | 0.22 |
| ⟨τ⟩ (ns) | 8.6 | 10.95 | 7 | 6 | 1.5 |
| PLQY (%) | 18.7 | 46.6 | 52.9 | 71.4 | 62.4 |
| k_r_ (ns^-1^) | 0.125 | 0.077 | 0.074 | 0.065 | 0.072 |
| k_nr_ (ns^-1^) | 0.54 | 0.089 | 0.067 | 0.026 | 0.043 |

Table S5. Energy transfer efficiency of CsPbCl_3_: K^+^ (15%) PeNCs with increasing the Eu^3+^ ion-concentration.

| Eu^3+^ concentration (mol%) | 0 | 0.8 | 3.5 | 6.5 | 7.1 |
| --- | --- | --- | --- | --- | --- |
| Lifetime of exciton (ns) | 10.9 | 7.9 | 3.6 | 2.7 | 2.9 |
| η (%) | _ | 27.5 | 67 | 75.0 | 73.5 |

Table S6. The electron and hole defect densities of the PeNC film with various CP concentrations treated.

| Sample | V_TFL_ (e) V | V_TFL_ (h) V | Nt (e) cm^-3^ | Nt (h) cm^-3^ |
| --- | --- | --- | --- | --- |
| 0 mg mL^-1^ | 1.04 | 1.01 | 4.92×10^16^ | 4.77×10^16^ |
| 1.0 mg mL^-1^ | 0.70 | 0.92 | 3.31×10^16^ | 4.35×10^16^ |
| 2.0 mg mL^-1^ | 0.64 | 0.84 | 3.02×10^16^ | 3.97×10^16^ |
| 3.0 mg mL^-1^ | 0.58 | 0.75 | 2.74×10^16^ | 3.54×10^16^ |
| 4.0 mg mL^-1^ | 0.59 | 0.75 | 2.79×10^16^ | 3.54×10^16^ |

Table S7. The electron and hole mobilities of the PeNC film with various CP concentrations treated.

| Sample | Electron mobility  (cm^2^ V^-1^ s^-1^) | Hole mobility  (cm^2^ V^-1^ s^-1^) |
| --- | --- | --- |
| 0 mg mL^-1^ | 3.1×10^-4^ | 2.1×10^-4^ |
| 1.0 mg mL^-1^ | 3.6×10^-4^ | 3.2×10^-4^ |
| 2.0 mg mL^-1^ | 4.0×10^-4^ | 3.4×10^-4^ |
| 3.0 mg mL^-1^ | 4.1×10^-4^ | 3.8×10^-4^ |
| 4.0 mg mL^-1^ | 3.7×10^-4^ | 3.4×10^-4^ |

Table S8. Performance of perovskite-based white LEDs.

| **Year** | **Device** | **Active layer** | **EQE (%)** | **L_max_ (cd m^-2^)** | **Ref** |
| --- | --- | --- | --- | --- | --- |
| **2017** | ITO/NiOx/PEDOT: PSS  /HFSO/CsPb(Br_1.5_I_1_._5_)/Ca/Al | HFSO/CsPb(Br_1.5_I_1.5_) | - | 1200 | ^1^ |
| **2017** | ITO/NiO_x_/CsPb(Br_x_/l_3-x_)  /MEH: PPV/TPBi/LiF/Al | MEH:PPV/CsPb(Brx/Cl_3-x_) | - | 105 | ^2^ |
| **2018** | Sapphire/p-GaN  /(Ni/Au)/nZnO/CsPbBr_3_/In | p-GaN//(Ni/Au)/nZnO//CsPbBr_3_ | 0.042 | 267 | ^3^ |
| **2018** | ITO/PEDOT: PSS/PA_2_CsPb_2_I_7_  /BIPO: PolyTPD/CsPb(Br,Cl)_3_ /TPBi/LiF/Al | PA_2_CsPb_2_I_7_/CsPb(Br,Cl)_3_ | 0.22 | - | ^4^ |
| **2019** | ITO/ZnO/PEI/ZnCdS/ZnS  /CsPb(Br_1.65_/I_1.35_)/TCTA/MoO_3_/ Au | PA_2_CsPb_2_I_7_/CsPb(Br,Cl)_3_ | 0.015 | 275 | ^5^ |
| **2019** | ITO/ZnO/PEI/Sm^3+^ doped CsPbCl_3_ PeNCs/TCTA /MoO_3_/Au | Sm^3+^doped CsPbCl_3_ PeNCs | 1.2 | 938 | ^6^ |
| **2020** | ITO/PEDOT: PSS/CsPb(Br_1−y_I_y_)_3_/CsPb(Br_1−x_Cl_x_)_3_/TPBi/LiF/Al | CsPb(Br_1−y_I_y_)_3_/CsPb(Br_1−x_Cl_x_)_3_ | 0.008 | 30 | ^7^ |
| **2021** | ITO/PEDOT:PSS/TFB/CsPbI_3_/TPBi/LiF/Al | CsPbI_3_ | 6.5 | 12200 | ^8^ |
| **2021** | ITO/PEDOT: PSS/PVK/ Cs_2_AgIn_0.9_Bi_0.1_C_l6_ /TPBi/LiF/Al | Cs_2_AgIn_0.9_Bi_0.1_C_l6_ | 0.08 | 158 | ^9^ |
| **2021** | ITO/ PEDOT: PSS/Cs_3_Cu_2_I_5_/CsCu_2_I_3_/TmPyPB/LiF/Al | Cs_3_Cu_2_I_5_/CsCu_2_I_3_ | 3.1 | 1570 | ^10^ |
| **2021** | ITO/NiO_x_/PVK/ PBABr_1.4_(Cs_0.7_FA_0.3_PbBr_3_)/TPBi/LiF/Al/Ag/LiF/ CsPbBrI_2_ | PBABr_1.4_(Cs_0.7_FA_0.3_PbBr_3_)/  CsPbBrI_2_ | 12.2 | 2000 | ^11^ |
| **2022** | ITO/ZnO/PEI/CsPbCl_3_: K^+^, Eu^3+^/TCTA/MoO_3_/A | CsPbCl_3_: K^+^, Eu^3+^ | 5.4 | 1678 | This work |

Table S9. The energy transfer efficiency of the samples with bandgap modulation.

| Sample | CsPbCl_3_ | Cs_0.95_K_0.05_PbCl_3_ | Cs_0.9_K_0.1_PbCl_3_ | Cs_0.85_K_0.15_PbCl_3_ | Cs_0.8_K_0.2_PbCl_3_ |
| --- | --- | --- | --- | --- | --- |
| η (Sm^3+^, %) | 30 | 35 | 54 | 74 | 55 |
| η (Ce^3+^, %) | 6.7 | 8.3 | 11.4 | 20 | 15 |
| Br- ion | CsPbCl_3_ | CsPbCl_2.5_Br_0.5_ | CsPbCl_2_Br | CsPbCl_1.5_Br_1.5_ | CsPbCl_2_Br |
| η (Er^3+^, %) | 8 | 11 | 21 | 36 | 24 |

**Reference**

1. Huang, C.Y. et al., Hybridization of CsPbBr_1.5_I_1.5_ perovskite quantum dots with 9,9-dihexylfluorene co-oligomer for white electroluminescence. *Organic Electronics* **2017,** *44*, 6-10.

2. Yao, E. P., et al. High-Brightness Blue and White LEDs based on Inorganic Perovskite Nanocrystals and their Composites. *Advanced Materials.* **2017,** *29* (23), 1606859.

3. Yue, W., et al. White LED based on CsPbBr_3_ nanocrystal phosphors via a facile two-step solution synthesis route. *Materials Research Bulletin* **2018,** *104*, 48-52.

4. Mao, J., et al. All-Perovskite Emission Architecture for White Light-Emitting Diodes. *ACS Nano* **2018,** *12* (10), 10486-10492.

5. Wang, C., et al. White light-emitting devices based on ZnCdS/ZnS and perovskite nanocrystal heterojunction. *Nanotechnology* **2019,** *30* (46), 465201.

6. Sun, R., et al. Samarium-Doped Metal Halide Perovskite Nanocrystals for Single-Component Electroluminescent White Light-Emitting Diodes. *ACS Energy Letters* **2020,** *5* (7), 2131-2139.

7. Yu, H., et al. Single-emissive-layer all-perovskite white light-emitting diodes employing segregated mixed halide perovskite crystals. *Chemical Science* **2020,** *11* (41), 11338-11343.

8. Chen, J., et al. Efficient and bright white light-emitting diodes based on single-layer heterophase halide perovskites. *Nature Photonics* **2021,** *15* (3), 238-244.

9. Zhang, Y., et al. Lead-free Double Perovskite Cs_2_AgIn_0.9_Bi_0.1_Cl_6_ Quantum Dots for White Light-Emitting Diodes. *Advanced Science* **2022,** *9* (2), 2102895.

10. Chen, H., et al. Efficient and bright warm-white electroluminescence from lead-free metal halides. *Nature Communications* **2021,** *12* (1), 1421.

11. Chen, Z., et al. Utilization of Trapped Optical Modes for White Perovskite Light-Emitting Diodes with Efficiency over 12%. *Joule* **2021,** *5* (2), 456-466.
