# Supplementary material for: Efficient single-component white light emitting diodes enabled by lanthanide ions doped lead halide perovskites via controlling Förster energy transfer and specific defect clearance
Source: Light Sci Appl. 2022 Dec 6;11:340. doi: 10.1038/s41377-022-01027-9 (PMC9722690; doi:10.1038/s41377-022-01027-9)
Supplement: Supplementary file 3 — Confidential Review [file 41377_2022_1027_MOESM3_ESM.pdf]

文章保密与版权转让证明

承　诺　书

此文章不涉密且不存在造假、抄袭、一稿多投等学术不端行为，特此承诺。

第一（通讯）作者签字：

2022 年 10 月 14 日

《Light: Science & Applications》编辑部：

我单位 孙蕊，周东磊，丁毓娇，王玥，王雨琪，庄歆萌，刘帅男，丁楠，王天源，徐文，宋宏伟 作者（需按正式发表文章署名顺序，填写全部作者姓名）为你刊撰写的文章（题目：Efficient Single-Component White Light Emitting Diodes Enabled by Lanthanide Ions Doped Lead Halide Perovskites via Controlling Förster Energy Transfer and Specific Defect Clearance），经审查，未发现该文章存在涉密内容和造假、抄袭、一稿多投等学术不端现象。该文章若存在涉密内容和造假、抄袭、一稿多投等学术不端问题，《Light: Science & Applications》编辑部无需承担任何责任。该文章一经录用，其数字化复制权、发行权、汇编权及信息网络传播权将转让予《Light: Science & Applications》编辑部。

导师（课题负责人）签字

单位盖章

2022 年 10 月 14 日
